# Glycoprotein PTGDS promotes tumorigenesis of diffuse large B-cell lymphoma by MYH9-mediated regulation of Wnt–β-catenin–STAT3 signaling

**DOI:** 10.1038/s41418-021-00880-2

**Published:** 2021-11-06

**Authors:** Shunfeng Hu, Shuai Ren, Yiqing Cai, Jiarui Liu, Yang Han, Yi Zhao, Juan Yang, Xiangxiang Zhou, Xin Wang

**Affiliations:** 1 Department of Hematology, Shandong Provincial Hospital, Cheeloo College of Medicine, Shandong University, 250021 Jinan, Shandong China; 2 Department of Hematology, Shandong Provincial Hospital Affiliated to Shandong First Medical University, 250021 Jinan, Shandong China; 3 School of Medicine, Shandong University, 250012 Jinan, Shandong China; 4 Shandong Provincial Engineering Research Center of Lymphoma, 250021 Jinan, Shandong China; 5 Branch of National Clinical Research Center for Hematologic Diseases, 250021 Jinan, Shandong China; 6 National Clinical Research Center for Hematologic Diseases, The First Affiliated Hospital of Soochow University, 251006 Suzhou, China

**Keywords:** Oncogenes, Ubiquitylation, Epigenetics, Protein-protein interaction networks, Prognostic markers

## Abstract

Glycoprotein prostaglandin D2 synthase (PTGDS) is a member of the lipocalin superfamily and plays dual roles in prostaglandins metabolism and lipid transport. PTGDS has been involved in various cellular processes including the tumorigenesis of solid tumors, yet its role in carcinogenesis is contradictory and the significance of PTGDS in hematological malignancies is ill-defined. Here, we aimed to explore the expression and function of PTGDS in diffuse large B-cell lymphoma (DLBCL), especially the potential role of PTGDS inhibitor, AT56, in lymphoma therapy. Remarkable high expression of PTGDS was found in DLBCL, which was significantly correlated with poor prognosis. PTGDS overexpression and rhPTGDS were found to promote cell proliferation. Besides, in vitro and in vivo studies indicated that PTGDS knockdown and AT56 treatment exerted an anti-tumor effect by regulating cell viability, proliferation, apoptosis, cell cycle, and invasion, and enhanced the drug sensitivity to adriamycin and bendamustine through promoting DNA damage. Moreover, the co-immunoprecipitation-based mass spectrum identified the interaction between PTGDS and MYH9, which was found to promote DLBCL progression. PTGDS inhibition led to reduced expression of MYH9, and then declined activation of the Wnt-β-catenin-STAT3 pathway through influencing the ubiquitination and degradation of GSK3-β in DLBCL. The rescue experiment demonstrated that PTGDS exerted an oncogenic role through regulating MYH9 and then the Wnt-β-catenin-STAT3 pathway. Based on point mutation of glycosylation sites, we confirmed the N-glycosylation of PTGDS in Asn51 and Asn78 and found that abnormal glycosylation of PTGDS resulted in its nuclear translocation, prolonged half-life, and enhanced cell proliferation. Collectively, our findings identified for the first time that glycoprotein PTGDS promoted tumorigenesis of DLBCL through MYH9-mediated regulation of Wnt-β-catenin-STAT3 signaling, and highlighted the potential role of AT56 as a novel therapeutic strategy for DLBCL treatment.

## Introduction

Diffuse large B-cell lymphoma (DLBCL), a highly aggressive and heterogeneous tumor, is the most common form of non-Hodgkin lymphoma [1]. With the development of novel targeted therapy, the majority of DLBCL patients could be cured. However, 40–50% of DLBCL patients still presented refractory or relapse and eventually died of disease progression [2]. Therefore, identifying more novel therapeutic targets is still needed for DLBCL treatment [3].

Lipocalin prostaglandin D synthetase (L-PGDS), also known as PTGDS, is located in human chromosome 9 (9q34.2~34.3), the region of the lipocalin family [4, 5]. PTGDS acts as a bifunctional protein that catalyzes PGD2 production and transports lipophilic substances [5]. AT56, an orally active and selective inhibitor of PTGDS, could inhibit PTGDS activity in a competitive manner (*K*_m_ = 14 μM). After synthesis and modification, glycoprotein PTGDS could be secreted outside the cell and dissolved in body fluid.

Recent studies have revealed that PTGDS was overexpressed in malignant melanomas [6], ovarian carcinoma [7], and hepatocellular adenoma [8]. In contrast, other researchers found that PTGDS was down-regulated and inhibited tumor progression in prostate tumors [9], lung tumors [10, 11], and gastric cancer [12]. Moreover, recent investigations showed that PTGDS acted as modulators of PPARγ [9, 13], MAPK [11], and STAT3 [12] pathways, which were associated with the pathogenesis of hematological malignancy. However, the role and mechanism of PTGDS in hematological malignancy, especially DLBCL, have not been reported.

Our present study aimed to explore the expression level and functional mechanism of PTGDS in DLBCL. Enhanced expression of PTGDS in DLBCL was discovered for the first time, which was associated with unfavorable therapeutic efficiency and poor prognosis. Moreover, in vitro and in vivo studies showed that PTGDS inhibition distinctly inhibited DLBCL progression through MYH9-mediated regulation of Wnt–β-catenin–STAT3 signaling. Besides, PTGDS inhibition enhanced the drug sensitivity of DLBCL cells by inducing DNA damage. The low glycosylation of PTGDS induced nuclear translocation, prolonged half-life, and increased cell proliferation in DLBCL. Altogether, our study demonstrated the oncogenic role and mechanism of glycoprotein PTGDS in DLBCL, highlighting the potential therapeutic value of AT56 in DLBCL treatment.

## Materials and methods

### Patient samples and cell lines

This study was approved by the Medical Ethical Committee of Shandong Provincial Hospital and written informed consent in accordance with the Declaration of Helsinki was obtained from each patient. Paraffin-embedded archived samples were collected from 120 newly diagnosed DLBCL patients and 32 reactive hyperplasia patients from 2012 to 2019. Histological diagnoses were established according to the 2016 WHO classification [14]. All enrolled DLBCL patients have received standard treatment according to international guidelines. Details of patients were provided in Supplementary Table 1. Peripheral blood mononuclear cells (PBMCs) and serum were isolated from 2017 and 2019. CD19^+^ B cells were purified from freshly isolated PBMCs [15]. LY1, LY3, LY8, VAL, and U2932 cells were purchased from ATCC, cultured in IMDM (Gibco, CA, USA) supplemented with 10% fetal bovine serum (HyClone, UT, USA), 1% penicillin/streptomycin mixture, and 2 mM glutamine, and incubated at 37 °C in humidified air containing 5% CO_2_. All cells were examined for short tandem repeat (STR) and mycoplasma infection periodically.

### Reagents

Recombinant human Wnt3a protein was obtained from R&D Systems (5036-WN, MN, USA) and recombinant human PTGDS protein (rhPTGDS) was bought from GenWay Biotech (GWB-AC3EE7, CA, USA). Adriamycin, bendamustine, and WP1066 were purchased from Selleck Chemicals (TX, USA). AT56 was from Cayman (13160, MI, USA) and Blebbistatin was bought from Abcam (ab120425, MA, USA). Tunicamycin (ab120296, Abcam) and PNGase F (P0704S, New England Biolabs, MA, USA) were purchased, respectively.

### Cell transfection

Lentivirus vectors encoding sh-PTGDS, LV-PTGDS, sh-MYH9, or control were from Genechem (Shanghai, China), and plasmids (His-PTGDS-WT, Flag-PTGDS-△51, HA-PTGDS-△78) were synthesized by Biosune (Shanghai, China).

### Immunohistochemistry (IHC) and hematoxylin–eosin (HE) staining

IHC and HE staining were performed according to standard methods. Staining results were evaluated by two independent observers who were blinded to patients’ clinical data at two different time points. IHC score was calculated by multiplying proportion score (0, none; 1, 1–25%; 2, 26–50%; 3, 51–75%; and 4, 76–100%) and intensity score (0, negative; 1, weak; 2, moderate; and 3, strong). Scores of 0–7 were defined as negative expression, 8–12 as positive expression. The primary antibodies included PTGDS (ab182141, Abcam), CD10 (ab256494, Abcam), and Ki67 (27309-1-AP, Proteintech Group).

### Western blotting

Total protein extraction and western blotting were performed following standard methods [15]. The nuclear and cytoplasmic proteins were extracted with NE-PER nuclear and cytoplasmic extraction reagents (Thermo Fisher Scientific, MA, USA). The primary antibodies included PTGDS (ab182141, Abcam; sc-390717, Santa Cruz Biotechnology), MYH9 (11128-1-AP, 60233-1-Ig, Proteintech Group), TRAF6 (66498-1-Ig, Proteintech Group), GSK3-β (22104-1-AP, Proteintech Group), and other antibodies bought from Cell Signaling Technology (Beverly, USA), including c-myc (18583), Cyclin D1 (2922), CDK2 (2546), caspase 3 (9662), caspase 9 (9508), PARP (9532), Bax (5023), Bcl-xl (2764), zeb-1(3396), vimentin (5741), P21 (2947), p-H2AX (Ser139, 9718), LRP6 (2560), p-LRP6 (Ser1490, 2568), p-GSK3-β (Ser9, 5558), β-catenin (8480), TCF4 (2569), p-STAT3 (Tyr705, 9145), STAT3 (9139), Ubiquitin (3936), His (12698), Flag (14793), and HA (3724). β-tubulin (86298), Histone H3 (4499), and GAPDH (97166) were served as the internal reference.

### Elisa assay

Serum was collected from 53 DLBCL patients and 17 healthy volunteers, and serum soluble PTGDS levels were measured using a human PTGDS ELISA Kit (MB-0408, MBBIOLOGY, China). Cell culture supernatants were harvested and the concentration of PGD2 was measured using human PGD2 ELISA Kit (MB-4041, MBBIOLOGY).

### Cell proliferation, viability, and invasion assay

Cell proliferation was assessed using the Cell Counting Kit-8 (CCK-8) assay kit (CK04, Dojindo, Japan) and Multiskan GO Microplate Reader (Thermo Scientific, IL, USA). Cell viability was evaluated using CellTiter-Glo Luminescent assays (G7570, Promega Corporation, WI, USA) and luminescence was recorded with a microplate luminometer (Centro XS3 LB960, Berthold Technologies, Stuttgart, Germany). Cell invasion was evaluated using 24-well transwells (8.0 μm, Corning, USA) precoated with matrigel. 600 μL IMDM with 10% FBS was added to the lower chamber, and 1 × 10^5^ treated cells suspended in 200 μL IMDM without FBS were seeded to the upper chamber and cultured at 37 °C for 24–48 h. The number of DLBCL cells in the lower chamber was counted using a cell counting plate.

### Flow cytometry analysis

Cell cycle and cell apoptosis were assessed by flow cytometry on Navios Flow Cytometer (Beckman Coulter, CA, USA) according to the manufacturer’s instructions.

### Comet assay

Alkaline single-cell gel electrophoresis assay was performed according to the protocol from Trevigen (4250-050-K). Slides were stained with DAPI and images were acquired using Olympus (IX73) inverted microscope. At least 65 cells per group were analyzed using CASP comet software.

### Co-immunoprecipitation and mass spectrometry

Protein extraction and purification were performed using Pierce™ Co-Immunoprecipitation Kit (26149, Thermo Fisher Scientific). Mass spectrometry was performed by Novogene (Beijing, China) and results were provided in Supplementary Table 3. The primary antibodies included PTGDS (ab182141, Abcam), MYH9 (60233-1-Ig, Proteintech Group), and GSK3-β (22104-1-AP, Proteintech Group).

### Cycloheximide (CHX) chase assay

DLBCL cells were incubated with 20 μM MG132 (HY-13259, MCE, USA), 10 μg/mL tunicamycin or left untreated. After the treatment of 50 μg/mL CHX (HY-12320, MCE) for a different time, cells were harvested and prepared for Western blotting.

### Immunofluorescence assay and confocal microscopy

Confocal microscopy was performed using the Leica TCS SP8 MP confocal microscope system (Germany). The primary antibodies included PTGDS (sc-390717, Santa Cruz Biotechnology), MYH9 (60233-1-Ig; 11128-1-AP, Proteintech Group), GSK3-β (22104-1-AP, Proteintech Group), p-H2AX (Ser139, 9718, CST), His (12698, CST) and Flag (14793, CST).

### Chromatin immunoprecipitation (ChIP) assay

ChIP assay was performed using Pierce Agarose ChIP Kit (26156, Thermo Scientific) according to the manufacturer’s instructions. The primary antibody was TCF4 (2569, CST). The primers of STAT3 were designed as described in a previous study [16].

### In vivo xenograft tumor models

This study was approved by the Animal Care and Research Advisory Committee of Shandong Provincial Hospital and guidelines of it were strictly followed in all animal experiments. No blinding was performed. The 4-week-old BALB/c nude male mice were randomized (simple randomization) into groups and 1 × 10^7^ LY1 cells (untransfected, empty control vector transfected, stably PTGDS-knockdown/overexpress vector transfected), were subcutaneously injected into their right hind legs. AT56 was dissolved in 0.5% methylcellulose and administered orally by gavage. The animals were imaged using an In-Vivo small animal imaging system (Berthold Technologies, Germany).

### Statistical analysis

All calculations were performed using SPSS 23.0 (SPSS Inc., USA). Each in vitro experiment was repeated three times and experimental data were depicted as mean ± standard deviation (SD). Data were tested for homogeneity of variances and normality. Quantitative variables were analyzed using Students *t*-test and non-parametric tests while categorical variables were analyzed by *χ*^2^-tests. Kaplan–Meier analysis was performed for survival curves and the difference between survival curves was compared using the Log-rank test. There was no statistical method used to determine the sample size in our study. Differences were considered statistically significant at *p* < 0.05 (*p < 0.05, **p < 0.01, ***p < 0.001).

## Results

### PTGDS was upregulated in DLBCL and associated with tumor progression

**Fig. 1:**
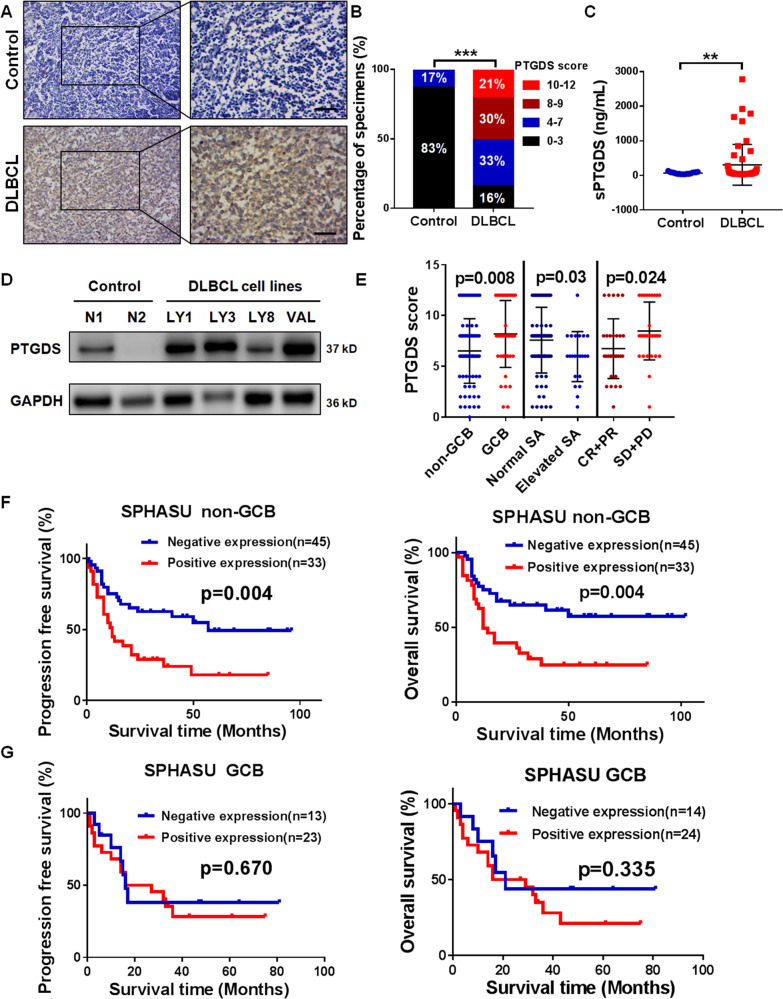
PTGDS was upregulated in DLBCL and associated with tumor progression. **A** and **B** Immunohistochemical staining for PTGDS expression was higher in DLBCL tissue (*n* = 120) than that in RHL samples (*n* = 32). Bar = 40 μm. **C** Higher serum PTGDS levels were found in DLBCL patients (*n* = 53) than healthy controls (*n* = 17). **D** Western blotting showed high expression of PTGDS in DLBCL cells. **E** Analysis of the association between clinical characteristics and PTGDS score in DLBCL. **F** and **G** Kaplan–Meier survival analysis of the association between PTGDS expression and PFS and OS in non-GCB and GCB patients, respectively. Data are shown as the mean ± SD. **p < 0.01; ***p < 0.001.

To elucidate the expression level of PTGDS in DLBCL, analysis based on IHC staining showed that PTGDS was upregulated in DLBCL tissues in comparison with control (Fig. 1A and B). Similarly, the level of serum PTGDS was higher (*p* < 0.01) in DLBCL patients (*n* = 53) than that in healthy control (*n* = 17) (Fig. 1C). Furthermore, compared with CD19^+^ B cells from PBMCs of healthy donors, the expression of PTGDS was increased in DLBCL cell lines (Fig. 1D).

The above findings prompted us to further evaluate the clinical significance of PTGDS in DLBCL patients. High expression of PTGDS was statistically correlated with GCB subtype, elevated sialic acid (SA), and unfavorable therapeutic efficacy in DLBCL patients (Fig. 1E, Supplementary Table 2). Although the expression of PTGDS was higher in the GCB subtype than that in the non-GCB subtype, PTGDS expression was negative in the normal germinal center (Supplementary Fig. S1A), indicating the specific high expression of PTGDS in lymphoma cells. Kaplan–Meier survival curve analysis (Supplementary Fig. S1B) indicated that in our study, DLBCL patients with positive PTGDS displayed reduced progression-free survival (29 months vs. 55 months, *p* = 0.003) and overall survival (31 months vs. 62 months, *p* = 0.001). Due to the correlation between disease subtype and prognosis in DLBCL [17], we further explored the prognostic value of PTGDS expression in GCB- and non-GCB subtypes separately. Interestingly, positive PTGDS expression was significantly correlated with worse prognosis in the non-GCB subtype (*p* < 0.01, Fig. 1F), but no significant difference was found in the GCB subtype (*p* > 0.05, Fig. 1G). These results indicated the potential role of PTGDS in the prognostic prediction of DLBCL patients, especially in the non-GCB subtype.

### PTGDS regulated the viability, proliferation, cell cycle, apoptosis, and invasion of DLBCL cells

**Fig. 2:**
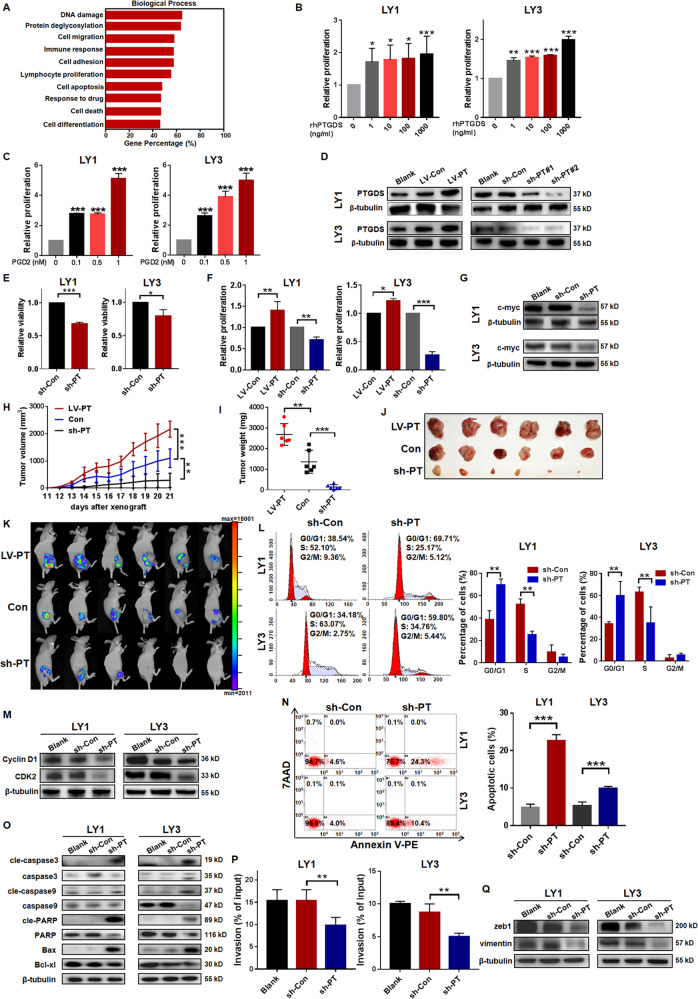
PTGDS regulated the viability, proliferation, cell cycle, apoptosis, and invasion of DLBCL cells. **A** GO analysis of PTGDS associated genes based on the GEO database (GSE31312). **B** and **C** The treatment with rhPTGDS and PGD2 promoted cell proliferation in a dose-dependent manner. **D** Relative expression of PTGDS protein was confirmed by western blotting in lentivirus transfected cells. **E**–**G** PTGDS overexpression promoted cell proliferation, and PTGDS knockdown inhibited cell viability, proliferation, and c-myc expression. **H**–**K** In a xenograft DLBCL mouse model, the growth rate, weight, volume, and bioluminescence of tumor were higher in the LV-PTGDS group than sh-PTGDS group (*n* = 6 per group). **L** and **M** PTGDS knockdown induced cell cycle arrest at G0/G1 phase and inhibited the expression of Cyclin D1 and CDK2. **N** and **O** PTGDS knockdown increased cell apoptosis and regulated the expression of apoptosis-associated proteins. **P** and **Q** PTGDS knockdown suppressed cell invasion and the expression of zeb1 and vimentin. After culture for 24–48 h, the percentage of cells in the lower chamber to input cells represented the level of cell invasion. Data are shown as the mean ± SD. *p < 0.05; **p < 0.01; ***p < 0.001.

To illuminate the role of PTGDS in DLBCL progression, we analyzed the data from the GEO database (GSE31312, GPL570, *n* = 498). Gene ontology (GO) analysis revealed that PTGDS was closely related to biological processes involved in tumor progression, such as cell proliferation, apoptosis, migration, and so on (Fig. 2A). To validate the bioinformatics results, functional experiments were performed using rhPTGDS, PGD2, and lentivirus targeting PTGDS, respectively.

To explore the influence of PTGDS on cell proliferation, DLBCL cells were incubated with rhPTGDS and increased proliferation was observed in a dose-dependent manner (Fig. 2B). Similarly, PGD2, the catalytic product of PTGDS, promoted cell proliferation (Fig. 2C) in DLBCL. In addition, we performed loss- and gain-of-function assays to further investigate the role of PTGDS in DLBCL. The effective regulation of PTGDS expression was demonstrated by western blotting (Fig. 2D), with sh-PTGDS#2 exhibiting higher efficacy of PTGDS knockdown. Cell viability was found to be suppressed by PTGDS knockdown in DLBCL (Fig. 2E). PTGDS knockdown decreased cell proliferation while PTGDS overexpression enhanced it (Fig. 2F). Furthermore, we found the decreased expression of pro-proliferation protein c-myc in sh-PTGDS cells (Fig. 2G).

To further verify the biological function of PTGDS in vivo, a mouse xenograft model was constructed (*n* = 6 per group). Mice bearing LV-PTGDS cells displayed increased growth rate (Fig. 2H), higher tumor volume and weight (Fig. 2I and J) at the end of the experiment. Furthermore, the bioluminescence of tumors based on the in-vivo imaging system was higher in mice bearing LV-PTGDS cells (Fig. 2K). Conversely, a significant reduction in tumor growth and bioluminescence was found in mice bearing sh-PTGDS cells (Fig. 2H–K). Besides, the expression of proliferation-related protein Ki67 was inhibited by PTGDS knockdown while LV-PTGDS increased it (Supplementary Fig. S2A). Altogether, these results indicated that PTGDS could promote cell proliferation in DLBCL.

PTGDS knockdown was observed to induce an obvious increment of cell proportion in the G0/G1 phase (*p* < 0.01), with concomitant decreases in the S phase (*p* < 0.01, Fig. 2L). In addition, the expression of Cyclin D1 and CDK2 was reduced by PTGDS knockdown (Fig. 2M), which promoted the transformation of the cell cycle from the G1 phase to the S phase. We further analyzed the regulatory effect of PTGDS on cell apoptosis and found that PTGDS knockdown increased cell apoptosis rates (Fig. 2N). After the knockdown of PTGDS, the expression of pro-apoptotic proteins (Bax, and the cleaved forms of caspase-3, caspase-9, and PARP) was increased, while the expression of anti-apoptotic protein Bcl-xl was reduced (Fig. 2O). Furthermore, transwell assays (Fig. 2P) showed a significant reduction of cell invasion in sh-PTGDS cells (*p* < 0.01). The expression level of zeb1 and vimentin, important positive factors of cell invasion, was decreased by PTGDS knockdown (Fig. 2Q). Taken together, our findings indicated that PTGDS might contribute to DLBCL progression through regulating viability, proliferation, cell cycle, apoptosis, and invasion.

### Targeted inhibition of PTGDS by AT56 displayed anti-tumor effects in DLBCL

**Fig. 3:**
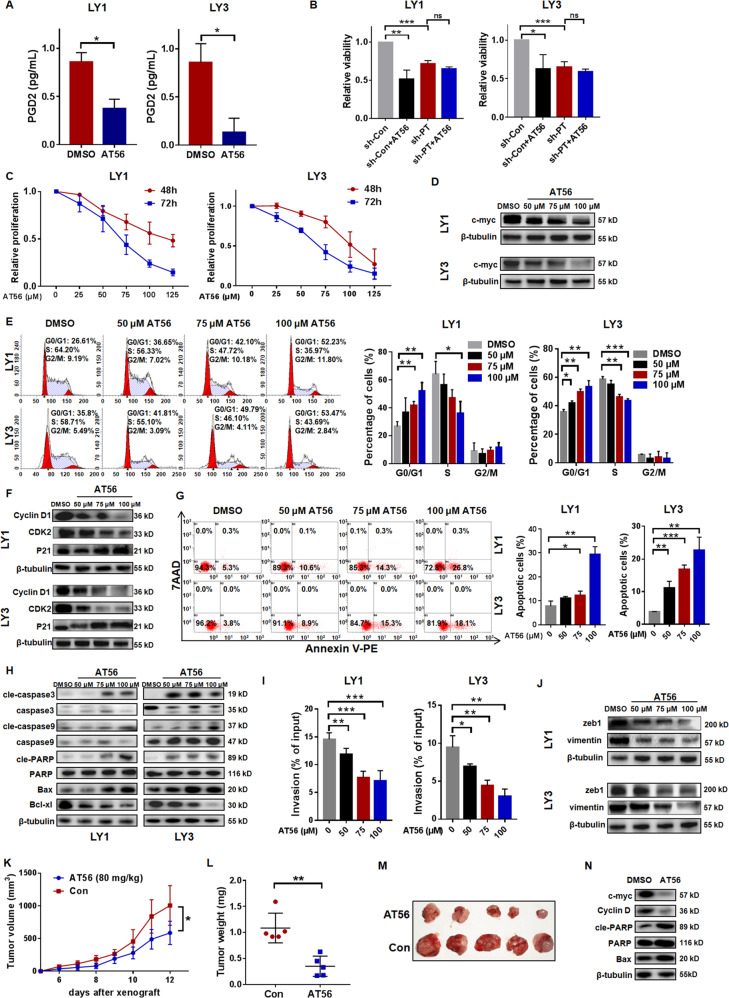
Targeted inhibition of PTGDS by AT56 displayed anti-tumor effects in DLBCL. **A** AT56 inhibited the production of PGD2 in DLBCL cells. **B** AT56 suppressed cell viability in sh-Con cells, but not in sh-PTGDS cells. **C** and **D** AT56 decreased cell proliferation and c-myc expression in DLBCL cells. **E** and **F** AT56 induced G0/G1 cell cycle arrest and decreased expression of Cyclin D1 and CDK2 in DLBCL cells. G-H. AT56 induced cell apoptosis and regulated the expression of apoptosis-associated proteins. **I** and **J** AT56 suppressed cell invasion and the expression of zeb1 and vimentin. **K**–**M** Nude mice with AT56 treatment (80 mg/kg) displayed decreased tumor growth rate, weight, and volume. **N** AT56 treatment regulated the expression of important proteins in mice tumor tissues. Data are shown as the mean ± SD. *p < 0.05; **p < 0.01; ***p < 0.001.

To further explore the role of PTGDS, we examined the effect of AT56, a selective inhibitor of PTGDS, in DLBCL cells. The concentration of PGD2 in the cell culture supernatant was decreased by AT56 (Fig. 3A). AT56 was found to inhibit cell viability in sh-Con cells, but not in sh-PTGDS cells (Fig. 3B), supporting the specificity of AT56 on PTGDS in DLBCL cells. Besides, cell proliferation was reduced by the incubation with AT56 in a dose-dependent and time-dependent manner (Fig. 3C). Dose-dependent reduction of c-myc was found in DLBCL cells treated with AT56 (Fig. 3D), which indicated the negative effect of AT56 on cell proliferation in DLBCL.

In addition, AT56 induced the elevation of G0/G1 phase cells in a dose-dependent manner (Fig. 3E). After AT56 treatment, the expression of Cyclin D1 and CDK2 decreased, and the expression of P21 increased, both in a dose-dependent manner (Fig. 3F). Flow cytometry demonstrated that AT56 dose-dependently increased the early apoptotic cell populations (Fig. 3G). Notably, the expression of pro-apoptotic proteins (Bax, and the cleaved forms of caspase-3, caspase-9, and PARP) was upregulated, and the expression of anti-apoptotic protein Bcl-xl was decreased with an elevated concentration of AT56 (Fig. 3H). The negative effect of AT56 on cell invasion was demonstrated and AT56 was observed to dose-dependently decrease the expression of zeb1 and vimentin in DLBCL cells (Fig. 3I and J).

The anti-tumor effect of AT56 in DLBCL was further confirmed in vivo using a xenograft model (*n* = 5 per group). Compared with the control group, mice receiving AT56 displayed a reduced growth rate (Fig. 3K), tumor weight (Fig. 3L), and volume (Fig. 3M). Besides, tumors from mice receiving AT56 displayed decreased expression of Ki67 (Supplementary Fig. S3A), c-myc, and Cyclin D1, and increased expression of Bax and cleaved PARP (Fig. 3N). Taken together, AT56 exerted therapeutic potential in DLBCL via regulating cell viability, proliferation, cell cycle, cell apoptosis, and invasion.

### PTGDS inhibition enhanced chemo-sensitivity of DLBCL cells through promoting DNA damage

**Fig. 4:**
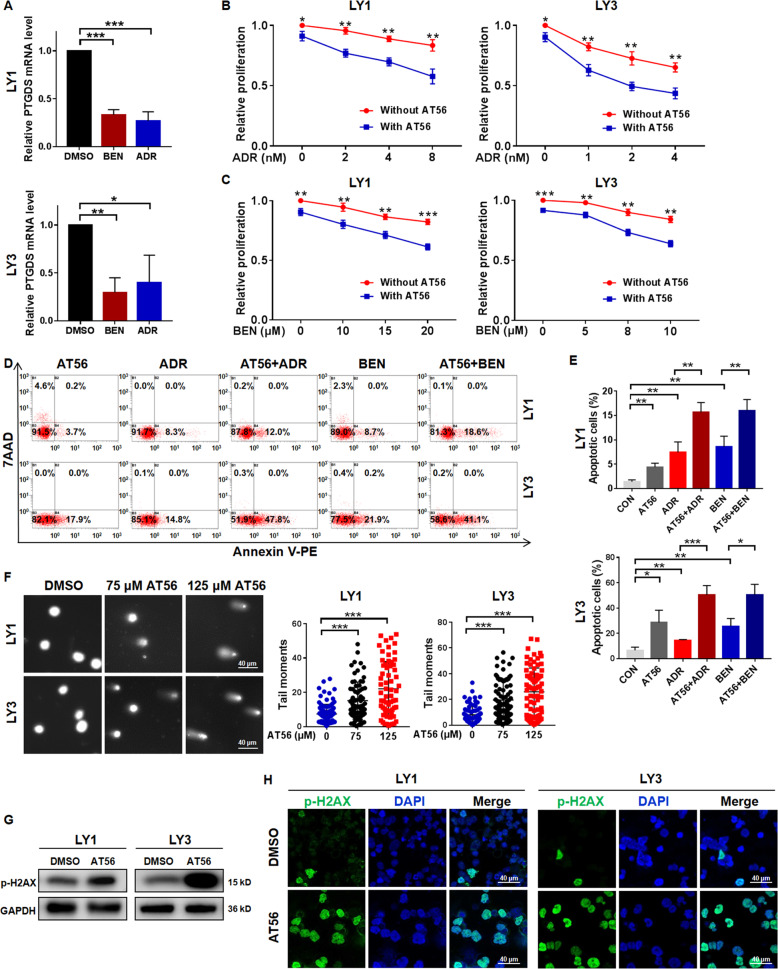
AT56 enhanced chemo-sensitivity of DLBCL cells through promoting DNA damage. **A** The expression level of PTGDS mRNA was reduced by adriamycin (ADR) and bendamustine (BEN). **B** and **C** The treatment of AT56 (50 μM) enhanced the cytotoxicity of ADR and BEN in LY1 and LY3 cells. **D** and **E** AT56 sensitized DLBCL cells to ADR and BEN in cell apoptosis. **F** Representative images and quantification of the tail of DLBCL cells treated with AT65 (75 and 125 µM) in the comet assay. Bar = 40 μm. **G** and **H** Western blotting and immunofluorescent images indicated that AT56 (75 μM) increased the expression of p-H2AX in DLBCL cells. Bar = 40 μm. Data are shown as the mean ± SD. *p < 0.05; **p < 0.01; ***p < 0.001.

To determine whether PTGDS was involved in drug sensitivity for DLBCL treatment, we firstly assessed the expression of PTGDS after drug treatment. It is found that adriamycin and bendamustine decreased the expression of PTGDS mRNA (Fig. 4A) in DLBCL cells. Notably, the addition of AT56 to adriamycin and bendamustine showed enhanced cytotoxicity in terms of cell proliferation (Fig. 4B and C) and apoptosis (Fig. 4D and E). Similarly, PTGDS knockdown also enhanced the drug sensitivity of DLBCL cells to adriamycin and bendamustine (Supplementary Fig. S4A and B).

As adriamycin and bendamustine exerted anti-tumor effects through promoting DNA damage, we further explored the effect of AT56 on DNA damage. Comet assay showed that DLBCL cells with AT56 treatment displayed longer tail moment (Fig. 4F), indicating enhanced DNA damage. Moreover, AT56 induced elevated expression of DNA damage marker (p-H2AX) in DLBCL cells (Fig. 4G and H), so as PTGDS knockdown (Supplementary Fig. S4C). Collectively, these findings provided evidence that PTGDS inhibition could sensitize DLBCL cells to chemotherapeutic drugs through promoting DNA damage, and further in-vivo studies were needed to confirm the effect of AT56 on chemo-sensitivity in DLBCL.

### MYH9 interacted with PTGDS and MYH9 inhibition displayed anti-DLBCL effects

**Fig. 5:**
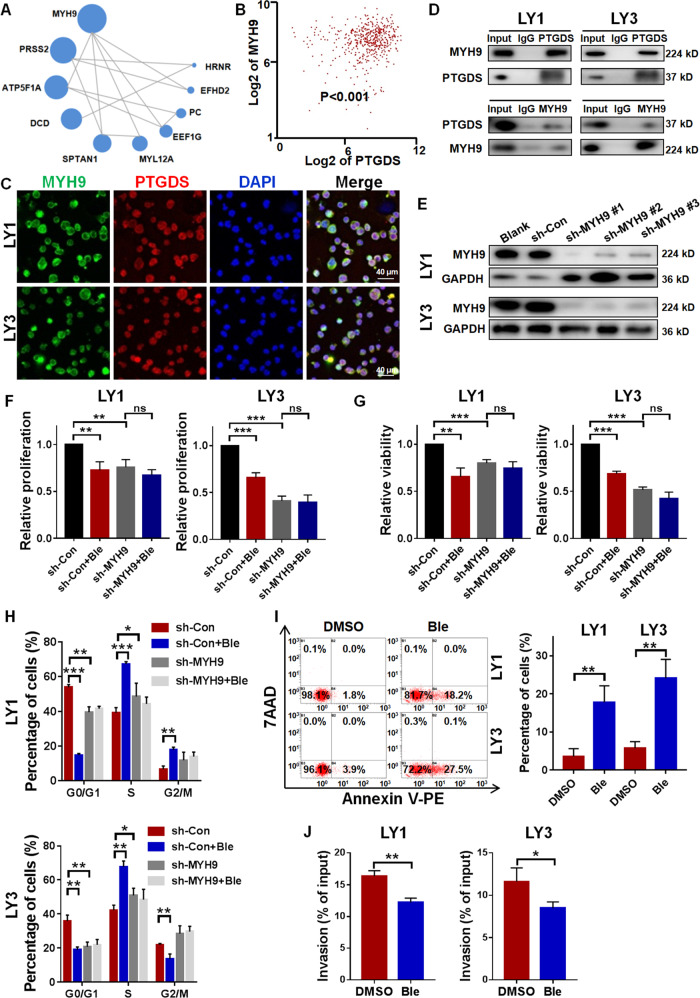
MYH9 interacted with PTGDS and MYH9 inhibition displayed anti-DLBCL effects. **A** Top 10 proteins in CoIP and mass spectrometry and their potential interactions. Circle size represented protein abundance. **B** Bioinformatic analysis based on GSE31312 indicated the association between the expression of PTGDS and MYH9. **C** Confocal immunofluorescent images indicated the colocalization of MYH9 and PTGDS protein in LY1 and LY3 cells. Bar = 40 μm. **D** CoIP assay showed the bindings between PTGDS protein and MYH9 protein in LY1 and LY3 cells. **E** Relative expression of MYH9 protein was confirmed by western blotting in lentivirus transfected cells. **F**–**J** MYH9 knockdown and Blebbistatin had effects on cell proliferation, viability, cell cycle, cell apoptosis, and cell invasion in DLBCL. Data are shown as the mean ± SD. *p < 0.05; **p < 0.01; ***p < 0.001.

To decipher the functional mechanisms of PTGDS in DLBCL, we performed CoIP and mass spectrometry and evaluated the PTGDS interactive proteins and possible regulatory pathways (Supplementary Table 3). The top 10 proteins and their potential interactions were presented in Fig. 5A and MYH9 ranked first among them. Bioinformatic analysis based on GSE31312 indicated the correlation between the expression level of PTGDS and MYH9 (*p* < 0.001) (Fig. 5B). Confocal immunofluorescent images illustrated the colocalization of PTGDS and MYH9 protein in DLBCL cells (Fig. 5C). Furthermore, CoIP and western blotting verified the interaction between endogenous PTGDS and MYH9 in DLBCL cells (Fig. 5D), indicating their interaction in natural conditions.

MYH9 has been reported to be involved in various human diseases, including chronic kidney disease, non-syndromic deafness, and tumor [18–20]. Given the interaction between PTGDS and MYH9, further experiments were performed to explore the role of MYH9 in DLBCL. Lentivirus-mediated RNA interference vectors against MYH9 displayed effective knockdown in DLBCL cells, of which sh-MYH9#1 exhibited the highest efficacy (Fig. 5E). MYH9 knockdown and MYH9 inhibitor, Blebbistatin, displayed anti-tumor effects in DLBCL through inhibiting cell proliferation and viability (Fig. 5F and G), inducing cell cycle arrest (Fig. 5H), promoting cell apoptosis (Fig. 5I), and decreasing cell invasion (Fig. 5J). In DLBCL cells with MYH9 knockdown, no effect of Blebbistatin on cell proliferation, viability, and cell cycle was found, supporting the specificity of Blebbistatin on MYH9 in DLBCL. Collectively, our results showed the interaction between PTGDS and MYH9 and the anti-tumor effects of MYH9 inhibition in DLBCL.

### PTGDS regulated MYH9 and then Wnt-β-catenin-STAT3 signaling through influencing the ubiquitination of GSK3-β in DLBCL

**Fig. 6:**
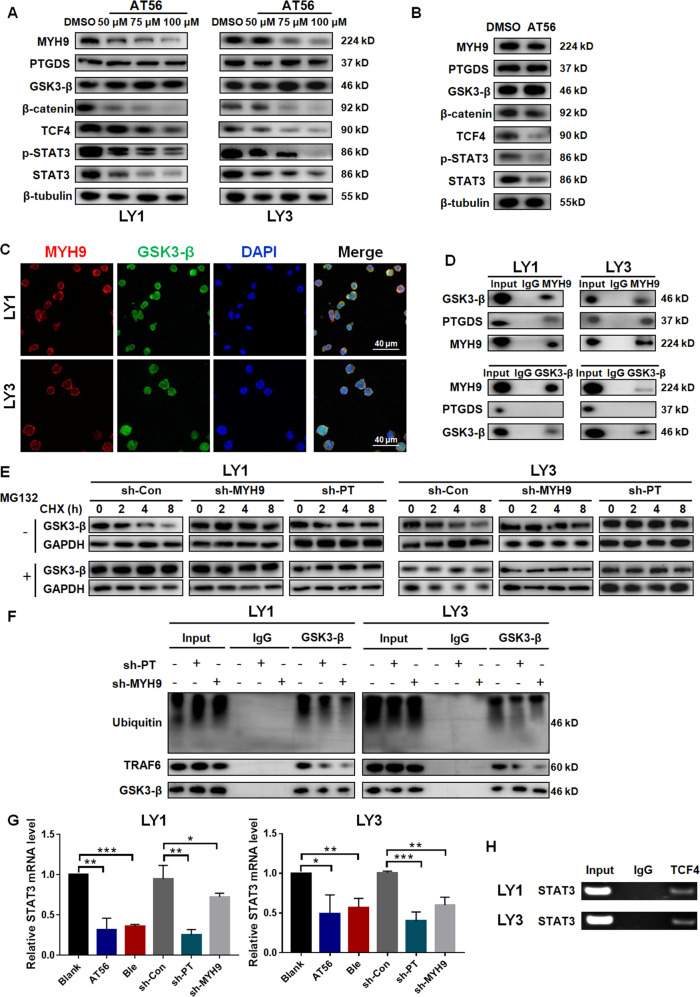
PTGDS regulated MYH9 and then Wnt–β-catenin–STAT3 signaling through influencing the ubiquitination of GSK3-β in DLBCL. **A** and **B** AT56 inhibited the expression of MYH9 and the activation of Wnt–β-catenin–STAT3 signaling in vitro and in vivo. **C** Confocal immunofluorescent images indicated the colocalization of MYH9 and GSK3-β protein in LY1 and LY3 cells. Bar = 40 μm. **D** CoIP assay showed the bindings between MYH9 protein and GSK3-β protein in LY1 and LY3 cells. **E** and **F** PTGDS and MYH9 inhibition decreased the ubiquitination of GSK3-β and prolonged its half-life in DLBCL. **G** The role of PTGDS and MYH9 inhibition on the expression of STAT3 mRNA in LY1 and LY3 cells. **H** ChIP assay showed the binding of TCF4 and STAT3 promoter in LY1 and LY3 cells. Data are shown as the mean ± SD. *p < 0.05; **p < 0.01; ***p < 0.001.

To further explore how PTGDS and MYH9 affected DLBCL progression, analysis based on mass spectrometry indicated that several PTGDS-associated proteins influenced the activation of the Wnt pathway (Supplementary Table 4), including MYH9. The Wnt pathway has been shown to play vital roles in hematological malignancies [21], especially in lymphoma. Several studies indicated that STAT3, an important transcription factor in tumors, was downstream of the Wnt pathway [16, 22, 23] and our previous research found that STAT3 inhibitor WP1066 exhibited anti-tumor effects in lymphoma [24]. Our results showed that AT56 (Fig. 6A and Supplementary Fig. S6A) and PTGDS knockdown (Supplementary Fig. S6B) decreased the expression of MYH9 and STAT3, and suppressed the activation of the Wnt pathway. Consistent results were also found in tumor tissues from mice receiving AT56 (Fig. 6B) or bearing sh-PTGDS cells (Supplementary Fig. S6C). However, no significant difference was found in sh-PTGDS cells (Supplementary Fig. S6D), supporting the specificity of AT56 on PTGDS in DLBCL again. Besides, MYH9 knockdown could also inhibit the activation of the Wnt pathway and the expression of STAT3 (Supplementary Fig. S6E).

MYH9 has been found to activate the Wnt pathway through enhancing transcription of β-catenin [25] or inducing the ubiquitination of GSK3-β [26–28]. To explore how PTGDS and MYH9 influenced the Wnt pathway in DLBCL, we evaluated the expression level of β-catenin, the key molecule of the Wnt pathway, followed by PTGDS and MYH9 inhibition. It was found that PTGDS and MYH9 inhibition decreased the protein level of β-catenin (Fig. 6A, B, Supplementary Fig. S6A–C), but not its mRNA expression (Supplementary Fig. S7A), which indicated enhanced degradation of β-catenin. Previous studies found the interaction between MYH9 and GSK3-β in hepatocellular carcinoma [26] and nasopharyngeal carcinoma [27, 28], and GSK3-β could induce the degradation of β-catenin. Our results showed the colocalization and interaction between endogenous MYH9 and GSK3-β in DLBCL cells, but not between PTGDS and GSK3-β (Fig. 6C, D, Supplementary Fig. S7B, C). In addition, PTGDS and MYH9 inhibition prevented the ubiquitination of GSK3-β and prolonged its half-life in DLBCL (Fig. 6E and F), which could induce the degradation of β-catenin and inhibit the activation of downstream molecules. The Wnt pathway has been found to regulate the phosphorylation of STAT3 through the activation of Fyn [29], and the expression of STAT3 through TCF4 [16, 22]. Our study found that PTGDS and MYH9 inhibition decreased both the mRNA (Fig. 6G) and protein levels of STAT3 in DLBCL cells (Fig. 6A, B, Supplementary Fig. S6A–C, and E), indicating the interruption of the transcriptional process. Furthermore, the ChIP assay showed the binding of TCF4, a transcription factor in the Wnt pathway, and STAT3 promoter in DLBCL cells (Fig. 6H and Supplementary Fig. S7E), indicating STAT3 as the downstream of Wnt pathway in DLBCL. Taken together, PTGDS was capable to regulate the expression of MYH9, thereby activating the Wnt–β-catenin–STAT3 pathway through influencing the ubiquitination of GSK3-β.

In addition, we found that PTGDS inhibition and MYH9 knockdown decreased the phosphorylation of LRP6 and GSK3-β (Supplementary Fig. S6F–H). In the canonical Wnt pathway, the decreased phosphorylation of GSK3-β could promote the ubiquitination and subsequent degradation of β-catenin, and then the inhibition of downstream molecules. Our findings indicated that PTGDS and MYH9 participated in the ligand-driven stimulation of the Wnt pathway, and further studies are still needed to illuminate the underlying mechanism.

### PTGDS promoted DLBCL tumorigenesis by MYH9-mediated regulation of Wnt–β-catenin–STAT3 signaling

**Fig. 7:**
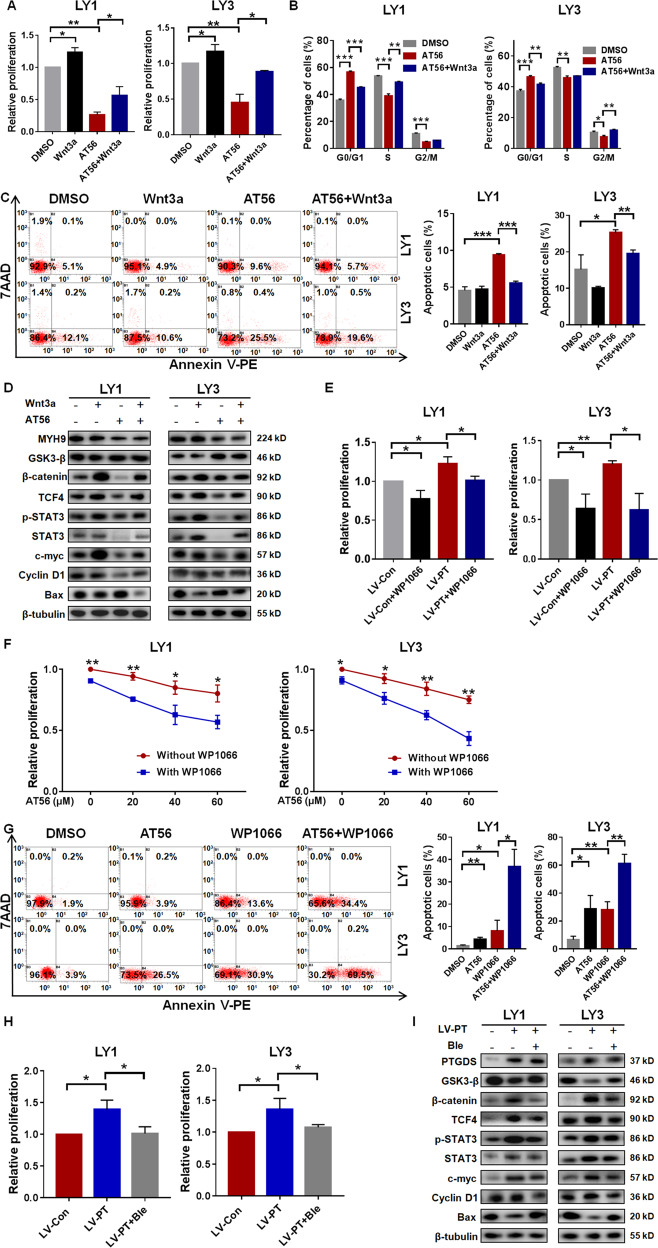
PTGDS promoted DLBCL tumorigenesis by MYH9-mediated regulation of Wnt–β-catenin-STAT3 signaling. **A** Wnt3a impaired the cytotoxicity of AT56 in LY1 and LY3 cells. **B** and **C** Wnt3a rescued the cell cycle arrest and cell apoptosis promotion caused by AT56. **D** The inhibition of the Wnt–β-catenin-STAT3 pathway by AT56 was reversed with Wnt3a. **E** WP1066 reversed the proliferation promotion caused by PTGDS overexpression. **F** WP1066 enhanced the effect of AT56 on cell proliferation in LY1 cells (2.5 μM) and LY3 cells (1 μM). **G** WP1066 enhanced the effect of AT56 on cell apoptosis in LY1 cells and LY3 cells. **H** and **I** Blebbistatin reversed the increased proliferation and the activation of Wnt–β-catenin–STAT3 signaling caused by PTGDS overexpression without change of PTGDS expression. Data are shown as the mean ± SD. *p < 0.05; **p < 0.01; ***p < 0.001.

Due to the regulatory role of PTGDS on MYH9 and the Wnt–β-catenin–STAT3 pathway, we further investigated whether the regulation was responsible for the oncogenic effect of PTGDS in DLBCL. As expected, when Wnt signaling was activated by Wnt3a, the proliferation inhibition by AT56 was significantly reversed (Fig. 7A). Wnt3a also rescued the effect of AT56 on cell cycle arrest (Fig. 7B) and cell apoptosis (Fig. 7C). Besides, the effect of AT56 on the Wnt pathway and other significant regulatory proteins could be partly reversed by Wnt3a (Fig. 7D), indicating that AT56 exerted anti-DLBCL effects through inhibiting the Wnt pathway. Moreover, the decreased expression of MYH9 after AT56 treatment could not be reversed by Wnt3a, suggesting that MYH9 might not act as the downstream of the Wnt pathway in DLBCL. Since STAT3 was downstream of the Wnt pathway, our data revealed that WP1066 reversed the proliferation promotion induced by PTGDS overexpression (Fig. 7E) and enhanced the proliferation inhibition (Fig. 7F) and apoptosis promotion (Fig. 7G) caused by AT56, indicating the involvement of STAT3 in the oncogenic role of PTGDS in DLBCL.

To explore whether PTGDS regulated Wnt pathway and DLBCL progression through MYH9, our data showed that enhanced cell proliferation caused by PTGDS overexpression was restored by Blebbistatin (Fig. 7H), indicating the involvement of MYH9 in the oncogenic role of PTGDS in DLBCL. Moreover, Blebbistatin restored the activation of the Wnt–β-catenin–STAT3 pathway caused by PTGDS overexpression (Fig. 7I), but with no change of PTGDS expression. Altogether, these data indicated that PTGDS could promote DLBCL progression through MYH9-mediated regulation of Wnt–β-catenin–STAT3 signaling.

### PTGDS glycosylation influenced its half-life, intracellular localization, and oncogenic role in DLBCL

**Fig. 8:**
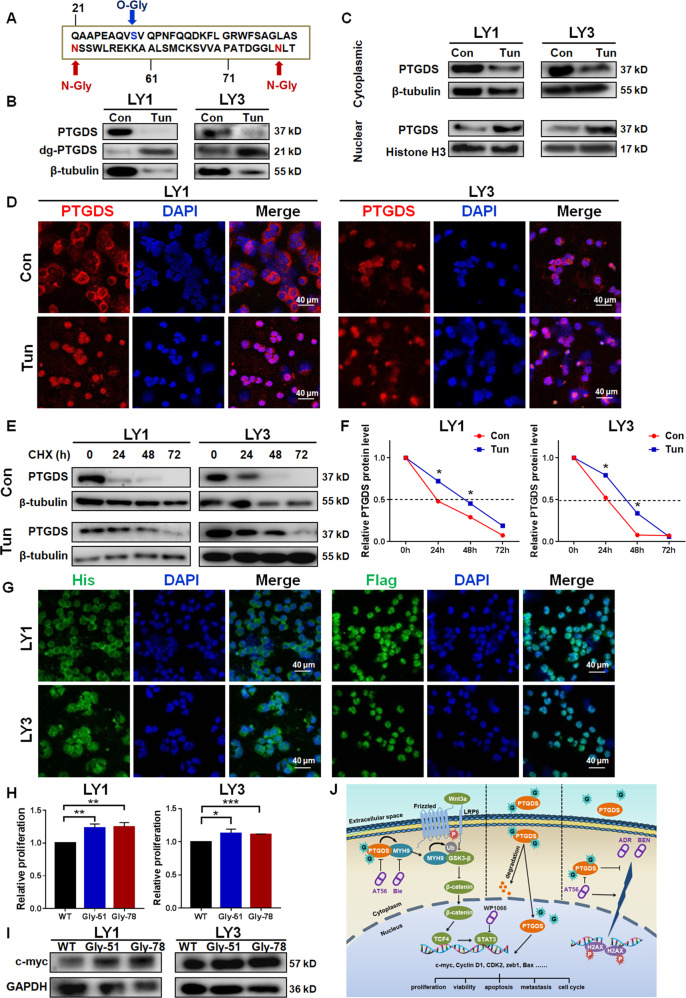
PTGDS glycosylation influenced its half-life, intracellular localization, and oncogenic role in DLBCL. **A** The glycosylation of PTGDS was predicted based on the Universal Protein Resource (UniProt) database. **B** The treatment of 10 μg/mL tunicamycin (Tun) increased the ratio of low molecular weight PTGDS protein. **C** and **D** Western blotting and immunofluorescent images indicated the nuclear translocation of PTGDS by Tun treatment. Bar = 40 μm. **E** and **F** Tun treatment inhibited PTGDS degradation and prolonged its half-life. **G** Glycosylation inhibition by point mutation (His-PTGDS-WT, Flag-PTGDS-△51) promoted nuclear translocation of PTGDS protein. Bar = 40 μm. **H** and **I** Glycosylation sites mutation promoted cell proliferation and the expression of c-myc in LY1 and LY3 cells. **J** Mechanism diagram summarized that glycoprotein PTGDS acted as a tumor enhancer through regulating the MYH9–Wnt–β-catenin–STAT3 axis in DLBCL. Data are shown as the mean ± SD. *p < 0.05; **p < 0.01; ***p < 0.001.

Protein glycosylation has been involved in tumor development and therapy [30]. We found the association between PTGDS expression and SA, the important terminal glycan in glycoprotein, in DLBCL. Besides, based on Universal Protein Resource (UniProt) database (Fig. 8A), it is found that PTGDS was with three specific glycosylation sites, including two N-glycosylation sites (Asn51 and Asn78) and an O-glycosylation site (Ser29). The removal of glycan chains using PNGase F (Supplementary Fig. S8A) and tunicamycin (Fig. 8B) increased the ratio of low molecular weight PTGDS protein, confirming the glycosyation of PTGDS in DLBCL. Furthermore, glycosylation inhibition by tunicamycin was found to promote the nuclear translocation of PTGDS in DLBCL (Fig. 8C and D).

PTGDS protein was upregulated in DLBCL, but its mRNA expression was decreased (Supplementary Fig. S8B). As glycosylation was known to modulate protein degradation, it promoted us to illuminate the role of glycosylation in PTGDS degradation in DLBCL. The glycosylation level of PTGDS in DLBCL cells was found to be lower than that in normal B cells (Supplementary Fig. S8C). In the CHX chase assay, tunicamycin led to degradation inhibition (Fig. 8E) and a longer half-life of PTGDS (Fig. 8F) in DLBCL, which might partly explain the discrepancy between PTGDS protein and mRNA expression level.

To further explore the glycosylation sites of PTGDS protein in DLBCL, point mutations were created in two potential N-glycosylation sites (Asn51 and Asn78). Mutated PTGDS displayed lower molecular weight than wild-type PTGDS (Supplementary Fig. S8D), which verified the glycosylation sites of PTGDS in DLBCL. Point mutation promoted the translocation of PTGDS to the nucleus (Fig. 8G), which was in consistent with the effect of tunicamycin. Furthermore, the deglycosylation by point mutation was found to promote cell proliferation (Fig. 8H) and increase the expression of c-myc (Fig. 8I) in DLBCL.

Collectively, PTGDS in DLBCL was with a low degree of glycosylation, and Asn51 and Asn78 were its N-glycosylation sites. Decreased glycosylation of PTGDS could induce nuclear translocation, prolong its half-life and promote cell proliferation, which might partly account for the oncogenic role of PTGDS in DLBCL.

## Discussion

In this study, our observations elucidated for the first time the high expression and oncogenic role of PTGDS in DLBCL. PTGDS was upregulated in human DLBCL and its high expression was correlated with inferior prognosis of DLBCL patients, especially non-GCB subtype. PTGDS inhibition displayed potential therapeutic effect in suppressing cell viability, proliferation, invasion, and promoting cell cycle arrest, apoptosis through MYH9-mediated regulation of Wnt–β-catenin–STAT3 signaling. The aberrant glycosylation of PTGDS promoted nuclear translocation, prolonged half-life, and increased cell proliferation in DLBCL. These inspiring findings have significant implications for the development of novel therapeutic strategies to promote the long-term survival of DLBCL patients.

As a PGD2-production enzyme and lipophilic ligands transporter, the role of PTGDS in cancer has not received much attention. Limited researches demonstrated that PTGDS was overexpressed in some solid tumors [6, 31], and its expression was correlated with advanced tumor stages, tumor metastasis [6], and poor prognosis [32]. Whereas, other studies identified that PTGDS was downregulated in prostate tumors [9], non-small cell lung cancer [10, 11], and gastric cancer [12]. Recently, bioinformatics analysis indicated that the expression of PTGDS mRNA was downregulated in DLBCL and displayed prognostic value [33]. However, there is no literature that explores the biological function and mechanism of PTGDS in hematological cancers, especially in DLBCL. We observed remarkably increased expression of PTGDS protein in DLBCL for the first time. It is found that the expression of PTGDS was higher in the GCB subtype, but there was no correlation between PTGDS expression and prognosis in the GCB subtype, which might be due to the small number of patients or homogeneity of high PTGDS expression in the GCB subtype. Furthermore, high PTGDS expression was significantly associated with inferior prognosis in non-GCB subtype and all DLBCL patients, and this consistency might be explained by the larger number of patients in the non-GCB subtype (*n* = 78) than GCB subtype (*n* = 42). Collectively, our findings indicate that PTGDS expression may be a potential prognostic predictor in non-GCB DLBCL patients, and further studies with more patients and longer follow-up are needed to explore its prognostic role in the GCB subtype.

Previous studies have found the anti-proliferation and anti-invasion effects of PTGDS in solid cancers, including prostate tumor [9], gastric carcinomas [12, 13], lung tumors [10, 11], testicular cancer [34], and melanoma [6, 31], but not in hematologic malignancies. Here, our study illuminated the cancer-promoting effects of PTGDS in DLBCL. PTGDS inhibition displayed the potency against DLBCL progression by viability and proliferation inhibition, apoptosis activation, G0/G1 cell cycle arrest, and cell invasion depletion. Our study in the DLBCL xenograft model also indicated the inhibitory effect of PTGDS inhibition on tumor progression. Taken together, our findings provide evidence for the oncogenic role of PTGDS in DLBCL and further studies will illuminate the role of PTGDS in other hematologic malignancies.

AT56 has been involved in several diseases processes and therapy, including neurogenic hypertension [35], metabolic syndrome [36], cardiovascular diseases [37], prostatic hyperplasia [38], and Crohn’s disease [39]. Nevertheless, the effect of AT56 in tumor therapy remains unexplored. In vivo and in vitro studies demonstrated the anti-tumor effect of AT56 in DLBCL and provided novel therapeutic options for DLBCL treatment. Drug resistance and toxicity could lead to worse clinical efficiency, and combination therapy has the potential to reduce drug dosages and toxicity, and decrease drug resistance. Our study found that PTGDS inhibition could sensitize DLBCL cells to adriamycin and bendamustine through promoting DNA damage, supporting the potential of AT56 in combination chemotherapy for DLBCL. Further research is required about the clinical use of AT56 in DLBCL treatment.

Previous investigations have implicated that PTGDS influenced tumor progression by modulating PPARγ [9, 13], MAPK [11], and STAT3 pathways [12]. Our results showed that PTGDS might promote DLBCL progression through MYH9-mediated regulation of Wnt–β-catenin–STAT3 signaling. Blebbistatin, an MYH9 inhibitor, functioned as a tumor suppressor in multiple cancers [40] and was demonstrated to inhibit DLBCL progression. There are several Wnt–β-catenin-targeted agents for the treatment of solid tumors and hematological malignancies in clinical trials [41]. Besides, WP1066, a STAT3 inhibitor, exhibited anti-cancer effects in multiple tumors [42, 43], including lymphoma [24], and we found WP1066 enhanced the inhibitory effects of AT56 on DLBCL progression. As PTGDS was demonstrated to regulate MYH9 and Wnt–β-catenin–STAT3 axis, further investigations are needed to explore the therapeutic effect of combination therapy among AT56, WP1066, Blebbistatin, and Wnt–β-catenin-targeted agents in DLBCL treatment.

Our study found that high expression of PTGDS protein in DLBCL was associated with worse prognosis and the inhibited tumor growth by PTGDS knockdown and AT56 supported the oncogenic role of PTGDS in DLBCL. However, the mRNA of PTGDS was found to be lower in DLBCL and Sun et al. indicated the low expression of PTGDS mRNA was correlated with a worse prognosis [33]. Post-transcriptional and post-translational modifications have effects on biological activity, subcellular location, and protein stability [44, 45]. Besides, abnormal glycosylation has been involved in several diseases [46] and recent studies found that glycosylation had important roles in lymphoma cell biology [47], therapy [48, 49], and prognosis [50]. Our results indicated the N-glycosylation sites of PTGDS (Asn51 and Asn78) in DLBCL, which was in agreement with the previous study [51]. The low glycosylation level of PTGDS in DLBCL was found to inhibit PTGDS degradation and prolong its half-life, which might partly explain the difference between the mRNA and protein level of PTGDS and their different prognostic significance in DLBCL. The abnormal glycosylation of PTGDS could enhance DLBCL cell proliferation, which might partly account for the oncogenic role of PTGDS in DLBCL and provide potential targets for lymphoma therapy. Further investigations on the detailed molecular mechanisms involved in PTGDS glycosylation in DLBCL development are still needed.

In summary, our results demonstrated for the first time the high expression and oncogenic role of PTGDS in DLBCL, and the therapeutic potency of AT56 in DLBCL treatment. Notably, PTGDS inhibition displayed excellent anti-lymphoma effects in vitro and in vivo study, through MYH9-mediated regulation of Wnt–β-catenin–STAT3 signaling. Besides, the abnormal glycosylation of PTGDS might influence its intracellular location, half-life, and cancer-promoting role. Collectively, our findings demonstrated glycoprotein PTGDS as a novel target for DLBCL treatment and highlighted the potency of AT56 as a promising therapeutic strategy for DLBCL.

## Supplementary information

Supplemental materials

Supplemental Table 1

Supplemental Table 2

Supplemental Table 3

Supplemental Table 4

## Data Availability

The datasets used and/or analyzed during the current study are available from the corresponding authors on reasonable request.
