# Supplementary material for: Glycoprotein PTGDS promotes tumorigenesis of diffuse large B-cell lymphoma by MYH9-mediated regulation of Wnt–β-catenin–STAT3 signaling
Source: Cell Death Differ. 2021 Nov 6;29(3):642–56. doi: 10.1038/s41418-021-00880-2 (PMC8901925; doi:10.1038/s41418-021-00880-2)
Supplement: Supplementary file 1 — Supplemental materials [file 41418_2021_880_MOESM1_ESM.docx]

Glycoprotein PTGDS **promotes tumorigenesis of** diffuse large B-cell lymphoma by MYH9-mediated regulation of Wnt-β-catenin-STAT3 signaling

Shunfeng Hu^1^, Shuai Ren^1^, Yiqing Cai^1^, Jiarui Liu^1^, Yang Han^1^, Yi Zhao^1^, Juan Yang^1^, Xiangxiang Zhou^1,2,3,4,5,6*^,Xin Wang^1,2,3,4,5,6*^

^1^ *Department of Hematology, Shandong Provincial Hospital, Cheeloo College of Medicine, Shandong University, Jinan, Shandong, 250021, China.*

^2^ *Department of Hematology, Shandong Provincial Hospital Affiliated to Shandong First Medical University, Jinan, Shandong, 250021, China.*

^3^ *School of Medicine, Shandong University, Jinan, Shandong, 250012, China.*

^4^ *Shandong Provincial Engineering Research Center of Lymphoma, Jinan, Shandong, 250021, China.*

^5^ *Branch of National Clinical Research Center for Hematologic Diseases, Jinan, Shandong, 250021, China.*

^6^ *National Clinical Research Center for Hematologic Diseases, the First Affiliated Hospital of Soochow University, Suzhou, 251006, China.*

**Running title:** Glycoprotein PTGDS promotes tumorigenesis of DLBCL

^*^**Corresponding authors**

Xin Wang, M.D., Ph.D.

Director & Professor of Department of Hematology,

Shandong Provincial Hospital, Cheeloo College of Medicine, Shandong University Add: No.324, Jingwu Road, Jinan, Shandong, 250021, China

Tel: 0086-531-68776358(B); 0086-13156012606(M)

Fax: 0086-531-87061197(B);

Email: [xinw007@126.com](mailto:xinw007@126.com)

Xiangxiang Zhou, M.D., Ph.D.

Department of Hematology, Shandong Provincial Hospital, Cheeloo College of Medicine, Shandong University

Add: No.324, Jingwu Road, Jinan, Shandong, 250021, China.

Tel: 0086-531-68776358(B); 0086-15866695595(M)

E-mail: [xiangxiangzhou@sdu.edu.cn](mailto:xiangxiangzhou@sdu.edu.cn)

**Conflict of Interest Disclosures**

The authors declare no competing financial interests.

Supplemental Materials and Methods

**Cell transfection**

Lentivirus vectors either encoding sh-PTGDS, LV-PTGDS, sh-MYH9 or control were from Genechem (Shanghai, China). The following RNAi sequences were used: sh-PTGDS 1#, CAGGGCTGAGTTAAAGGAGAA; sh-PTGDS 2#, GATAAGTGCATGACGGAACAA; sh-MYH9 1#, GCAAACCTCGAGAAGGCAA; sh-MYH9 2#, GAAGTCAGCTCCCTAAAGA; sh-MYH9 3#, GGCCAAACCTGCCGAATAA. Lentivirus transfection was carried out according to manufacturers’ instruction. The stably transfected cells were selected by 5 μg/mL puromycin (AMRESCO, USA). Infection efficiencies were evaluated using green fluorescent protein (GFP) through flow cytometry and validated by western blotting. The plasmids (His-PTGDS-WT, Flag-PTGDS-△51, HA-PTGDS-△78) were synthesized by Biosune (Shanghai, China) and all plasmid transfections were performed with Lipofectamine 2000 (Invitrogen, CA, USA) according to the manufacturer’s instructions.

**Quantitative real-time PCR**

Total RNA of samples was extracted by RNAiso Plus reagent (Takara, Dalian, China). Reverse transcription reaction was conducted with PrimeScript RT reagent kit with gDNA eraser (Takara). The mRNA expression level was detected using SYBR Green Master Mix (TaKaRa) in LightCycler 480II real-time PCR system (Roche, Basel, Swizerland). The primers were as follows: PTGDS forward, 5′-AAGGAGAAATTCACCGCCTTCTG-3′; PTGDS reverse, 5′-GAAGGAACAGAGCAGAGACATCCA-3′; CTNNB1 forward, 5′-GCTGCAACTAAACAGGAAGGG-3′; CTNNB1 reverse, 5′-CCCACTTGGCAGACCATCAT-3′; STAT3 forward, 5′-TGGAAATAATGGTGAAGGTGCTGA-3′; STAT3 reverse, 5′-CTCCATGTCAAAGGTGAGGGA-3′. Relative quantification was calculated using the 2−ΔΔCT method. Real-time PCR for each gene of each cDNA sample was assessed in triplicate.

**Immunohistochemistry (IHC) and hematoxylin-eosin (HE) staining**

The 4-μm-thick tissue sections were sliced from formalin-fixed, paraffin-embedded tissue blocks from patients. After deparaffinization and rehydration, antigen retrieval was performed using 0.01 mol/L sodium citrate buffer (pH 6.0) under high pressure. Samples were then blocked endogenous peroxidases with 3% solution of hydrogen peroxide, which followed by incubation with normal goat serum to block non-specific binding sites. Following this, samples were incubated with specific primary antibodies overnight at 4 °C. After 1× PBS rinses for 15 minutes, tissue sections were incubated with second antibody for 1 hour at 37°C, followed by further treatment with strept avidin-horseradish peroxidase complex (SABC) for 30 minutes and stained with 3,3′- diaminobenzidine tetrachlorhydrate dehydrate (DAB). Sections were counterstained with hematoxylin and mounted. Pictures of five microscopic fields were taken at ×400 magnification. Staining results were evaluated by two independent observers who were blinded to patients’ clinical data at two different time points. The IHC score was calculated by multiplying proportion score (0, none; 1, 1%~25%; 2, 26%~50%; 3, 51%~75%; and 4, 76%~100%) and intensity score (0, negative; 1, weak; 2, moderate; and 3, strong). Fresh mice subcutaneous tumors were fixed in 4% paraformaldehyde and embedded with paraffin. Sections with 5-μm thickness were cut for IHC and HE staining. The primary antibodies included PTGDS (ab182141, Abcam), CD10 (ab256494, Abcam) and Ki67 (27309-1-AP, Proteintech Group).

**Western blotting**

Protein extraction and western blotting were performed following standard methods. Total protein was collected from DLBCL cells or tumor tissue using lysis buffer with 1×phosphatase inhibitor cocktail (PhosSTOP, Roche, Basel, Switzerland). The concentration of protein was measured by BCA assay (Shenergy Biocolor). The nuclear and cytoplasmic proteins were extracted with NE-PER Nuclear and Cytoplasmic Extraction Reagents (Thermo Fisher Scientific, MA, USA). Protein extracts (30 μg) were then electrophoresed on SDS-polyacrylamide gel and then transferred to polyvinylidene fluoride (PVDF) membranes (Millipore, MA, USA), which were incubated with blocking solution (Tris-buffered saline containing 5% skim milk and 0.1% Tween 20) for 1 hour at room temperature. After incubation with indicated primary antibodies at 4℃ overnight, membranes were washed with TBS-T and then treated with HRP-conjugated secondary antibodies (Zhongshan Goldenbridge). After treatment with chemiluminescence detection reagent (Merck Millipore, MA, USA), chemiluminescent signals were detected by the Amersham Imager 600 imaging system (General Electric, USA). ImageJ software (NIH) was used to quantify the protein bands normalized to control. The primary antibodies included PTGDS (ab182141, Abcam; sc-390717, Santa Cruz Biotechnology), MYH9 (11128-1-AP; 60233-1-Ig, Proteintech Group), TRAF6 (66498-1-Ig, Proteintech Group), GSK3-β (22104-1-AP, Proteintech Group) and other antibodies bought from Cell Signaling Technology (Cell Signaling Technology, Beverly, USA), including c-myc (18583), Cyclin D1 (2922), CDK2 (2546), caspase 3 (9662), caspase 9 (9508), PARP (9532), Bax (5023), Bcl-xl (2764), zeb-1(3396), vimentin (5741), P21 (2947), p-H2AX (Ser139，9718), LRP6 (2560), p-LRP6 (Ser1490, 2568), p-GSK3-β (Ser9, 5558), β-catenin (8480), TCF4 (2569), p-STAT3 (Tyr705，9145), STAT3 (9139), Ubiquitin (3936), His (12698), Flag (14793), and HA (3724). β-tubulin (86298), Histone H3 (4499) and GAPDH (97166) were served as the internal reference.

**Cell proliferation, viability and invasion assay**

DLBCL cells (1×10^4^ cells/ 100μl/ well) with indicated treatment were seeded onto 96-well plates. Cell proliferation was assessed using the Cell Counting Kit-8 (CCK-8) assay kit (CK04, Dojindo, Japan) and Multiskan GO Microplate Reader (Thermo Scientific, IL, USA). Cell viability was evaluated using CellTiter-Glo Luminescent assays (G7570, Promega Corporation, WI, USA) and luminescence was recorded with microplate luminometer (Centro XS3 LB960, Berthold Technologies, Stuttgart, Germany). Cell invasion was evaluated using 24-well transwells (8.0 μm, Corning, USA) precoated with matrigel. 600 μL IMDM with 10% FBS was added to lower chamber, and 1 × 10^5^ treated cells suspended in 200 μL IMDM without FBS were seeded to upper chamber and cultured at 37 °C for 24 - 48 hours. The number of DLBCL cells in lower chamber was counted using cell counting plate.

**Flow cytometry analysis**

Cell cycle and cell apoptosis were assessed by flow cytometry on Navios Flow Cytometer (Beckman Coulter, CA, USA) according to the manufacturer’s instructions. In cell apoptosis assay, DLBCL cells with indicated treatments were harvested, washed twice with cold PBS and resuspended in 1× binding buffer, followed by the addition of 5 μL of Annexin V-PE and 5 μL of 7-AAD. After gentle vibration and incubation for 15-30 minutes at room temperature in the dark, cells were subjected to the flow cytometry. In cell cycle analysis, after being washed with PBS and fixed with 70% ethanol overnight at -20 ℃, DLBCL cells with indicated treatments were stained with PI/RNase Staining Buffer for 15-30 minutes and then subjected to the flow cytometry. Propidium iodide (PI, 550825) and Annexin V-PE/7-aminoactinomycin D (7AAD) apoptosis detection kit (559763) were purchased from BD Biosciences (MA, USA).

**Comet assay**

Alkaline single-cell gel electrophoresis assay was performed according to the protocol from Trevigen (4250-050-K). Firstly, 1×10^4^ DLBCL cells were seed into 96-well plates and incubated with AT56 for 48 hours. Cells were then harvested and washed with 1X PBS. Then, we mixed 50 μL of cell suspension with 500 μL comet LMAgarose and pipetted 50 µl onto CometSlide, which was incubated in the dark at 4 °C for 30 minutes. Next, comet slides were immersed in lysis buffer 4 °C overnight in the dark and then incubated in alkaline unwinding solution for 40 minutes at room temperature. Electrophoresis was run at 1 V/cm and 300 mA in ice-cold alkaline electrophoresis solution for 30 minutes. Slides were washed by distilled water and immersed in 70% ethanol for 5 minutes. Slides were stained with DAPI and images were acquired using Olympus (IX73) inverted microscope. At least 65 cells per group were analyzed using the CASP comet software.

**Co-immunoprecipitation and mass spectrometry**

Protein extraction and purification from lymphoma cells were performed using Pierce™ Co-Immunoprecipitation Kit (26149, Thermo Fisher Scientific). SDS-PAGE and coomassie brilliant blue R250 (Solarbio Science & Technology, Beijing, China) were used to stain the gel and mass spectrometry was performed by Novogene (Beijing, China). Data analysis and protein identification were done based on NCBI protein database. The primary antibodies included PTGDS (ab182141, Abcam), MYH9 (60233-1-Ig, Proteintech Group), GSK3-β (22104-1-AP, Proteintech Group).

**Immunofluorescence assays and confocal microscopy**

Lymphoma cells with designed treatment were transferred to a glass slide via cytospin. Cells were fixed by 4% paraformaldehyde for 15 minutes, permeabilized by 0.1% Triton X 100 for 10 minutes. Thereafter, slides were blocked with 5% normal goat serum for 1 hour and incubated with primary antibodies at 4 °C overnight. Next, further incubation with corresponding secondary antibodies was performed, and then slides were washed and mounted with DAPI. Confocal microscopy was performed using the Leica TCS SP8 MP confocal microscope system (Germany). The primary antibodies included PTGDS (sc-390717, Santa Cruz Biotechnology), MYH9 (60233-1-Ig; 11128-1-AP, Proteintech Group), GSK3-β (22104-1-AP, Proteintech Group), p-H2AX (Ser139，9718, CST), His (12698, CST) and Flag (14793, CST).

**Chromatin immunoprecipitation (ChIP) assay**

ChIP assay was performed using Pierce Agarose ChIP Kit (26156, Thermo Scientific) according to the manufacturer’s instructions. 2×10^6^ DLBCL cells were harvested and cross-linked using 1% formaldehyde. Chromatin was digested using Micrococcal Nuclease and then incubated with 8 μg anti-TCF4 antibody or Rabbit IgG overnight at 4℃. DNA was purified and used as template for PCR reactions. The primary antibody was TCF4 (2569, CST). The primers of STAT3 were designed as described in a previous study： forward (5′-GCTCACGCAGAAACTGAAGTT-3′); reverse (5′-TTGAGAGCCTCTTACCACG-3′).

***In vivo* xenograft tumor models**

This study was approved by the Animal Care and Research Advisory Committee of Shandong Provincial Hospital and guidelines of it were strictly followed in all animal experiments. No blinding was performed. BALB/c nude male mice of 4-week old were bought (Weitong Lihua Laboratory Animal Center, Beijing, China) and raised in a pathogen-free environment with sterile food and drinking water. The mice were randomized (simple randomization) into groups and 1 × 10^7^ LY1 cells (untransfected, empty control vector transfected, stably PTGDS-knockdown/overexpress vector transfected), were subcutaneously injected into their right hind legs. AT56 was dissolved in 0.5% methylcellulose and administered orally by gavage. The size of tumors was measured by digital caliper every day. The animals were imaged using *In-Vivo* small animal imaging system (Berthold Technologies, Germany). The volume of tumor was calculated by the equation V = (a × b^2^) × 0.5, where a was the largest dimension and b was the perpendicular diameter. After 1-3 weeks, mice were sacrificed and tumors were removed and weighed.

**Supplemental Figures**

**
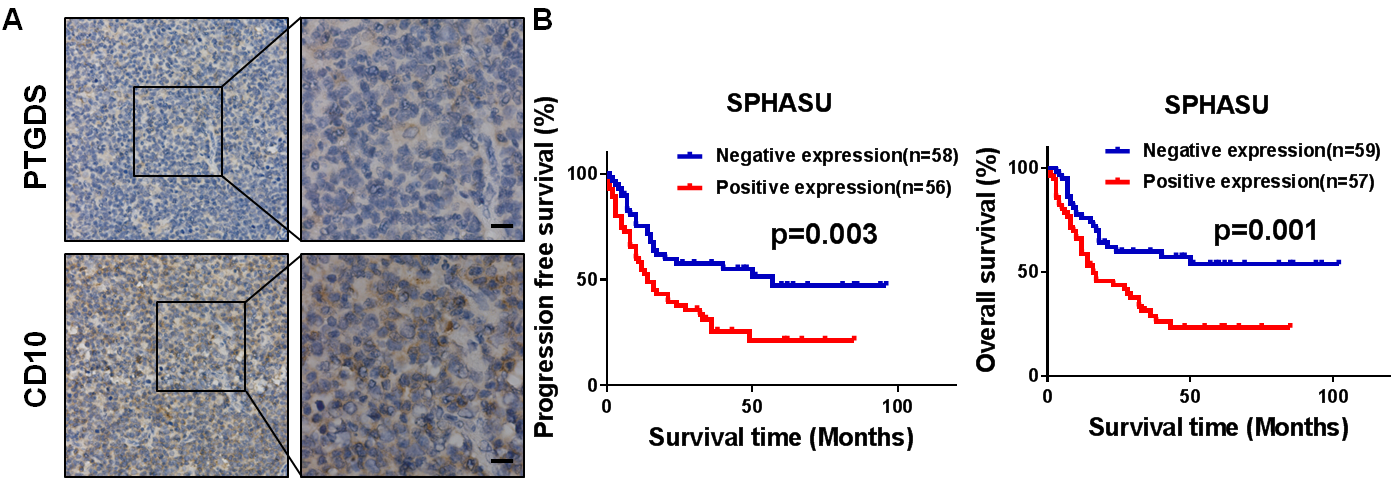
**

**Supplemental Figure 1. The association between PTGDS expression and prognosis in DLBCL patients.** A. Representative immunohistochemical images of germinal center in tonsil stained by PTGDS and CD10. Bar = 10 μm. B. Kaplan–Meier survival analysis of the association between PTGDS and PFS and OS in all enrolled DLBCL patients.


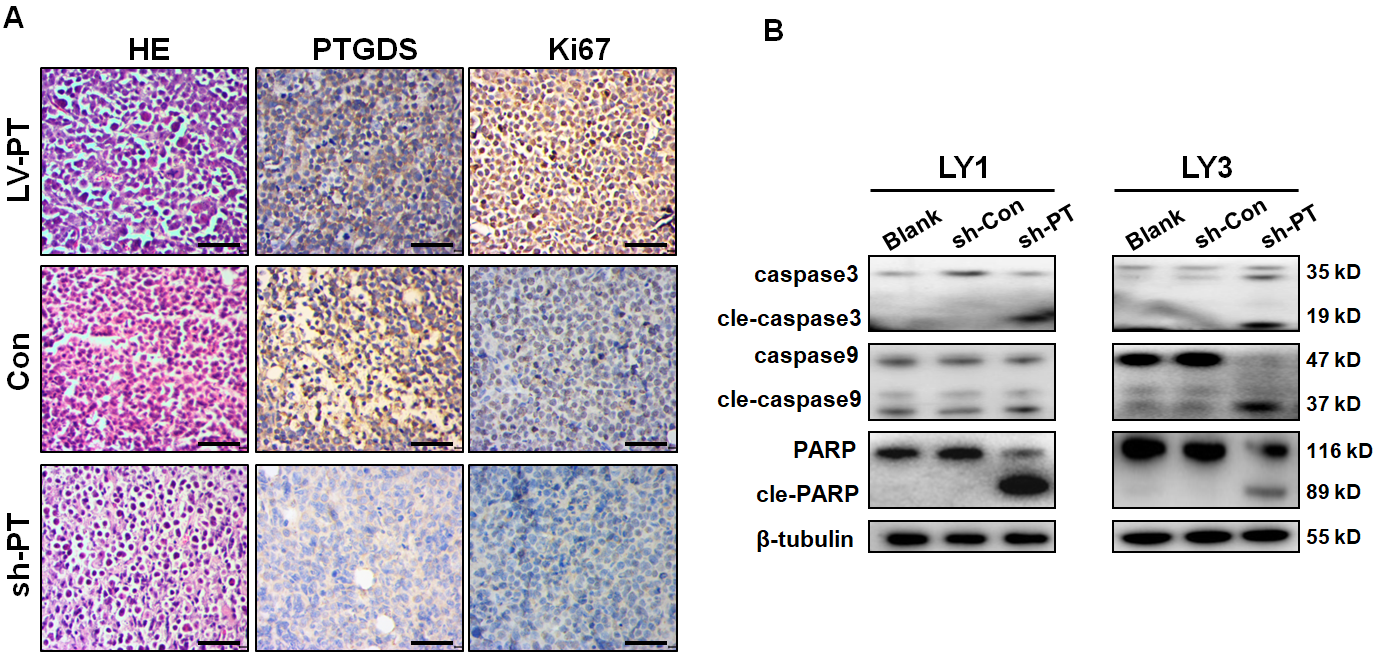


**Supplemental Figure 2. PTGDS knockdown inhibited cell proliferation *in vivo* and induced cell apoptosis*.*** A. Representative images of HE and IHC staining with PTGDS and Ki67 in xenograft tumor tissues. Bar = 50 μm. B. The whole blot of caspases and PARP expression in untransfected and transfected DLBCL cells.


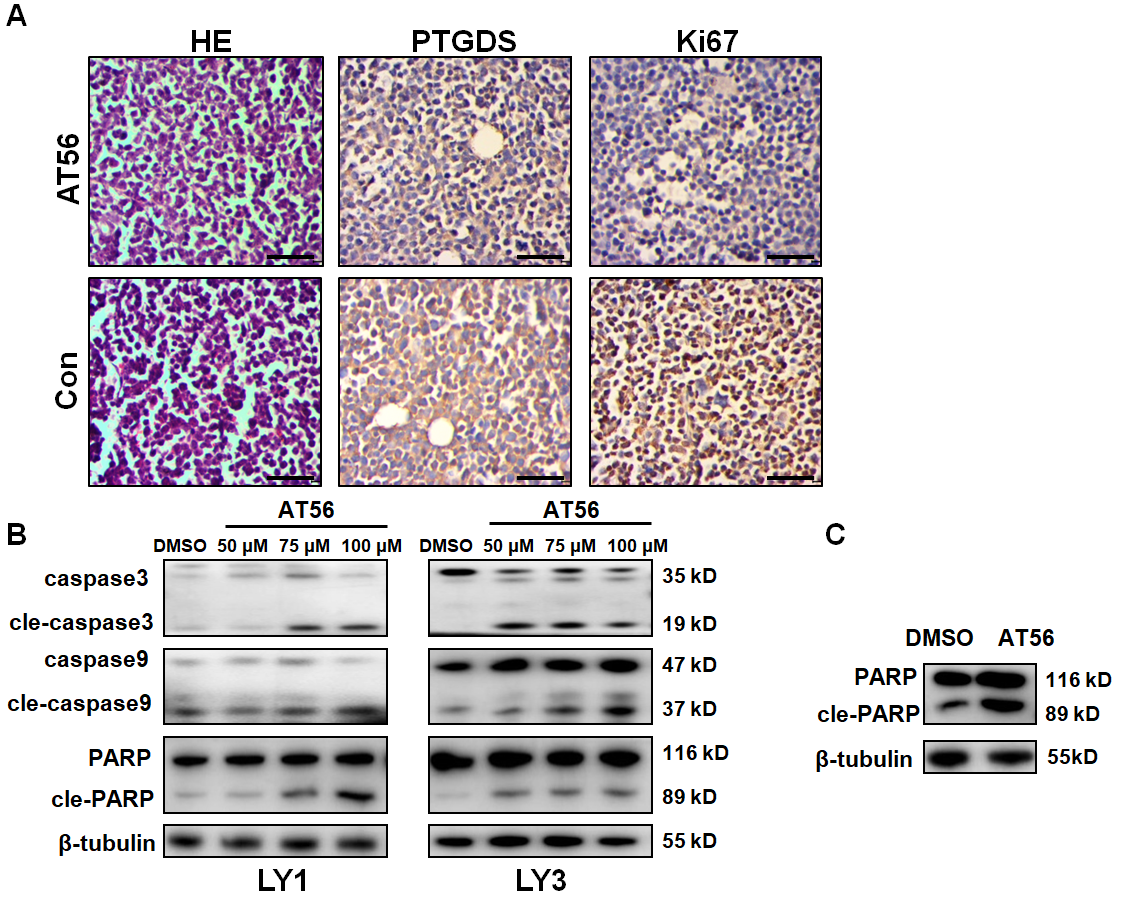


**Supplemental Figure 3. AT56 inhibited cell proliferation *in vivo* and induced cell apoptosis*.*** A. Representative images of HE and IHC staining with PTGDS and Ki67 in xenograft tumor tissues from mice receiving AT56 or control treatment. Bar = 50 μm. B-C. The whole blot of caspases and PARP expression in DLBCL cells with AT56 or control treatment *in vitro* and *in vivo*.


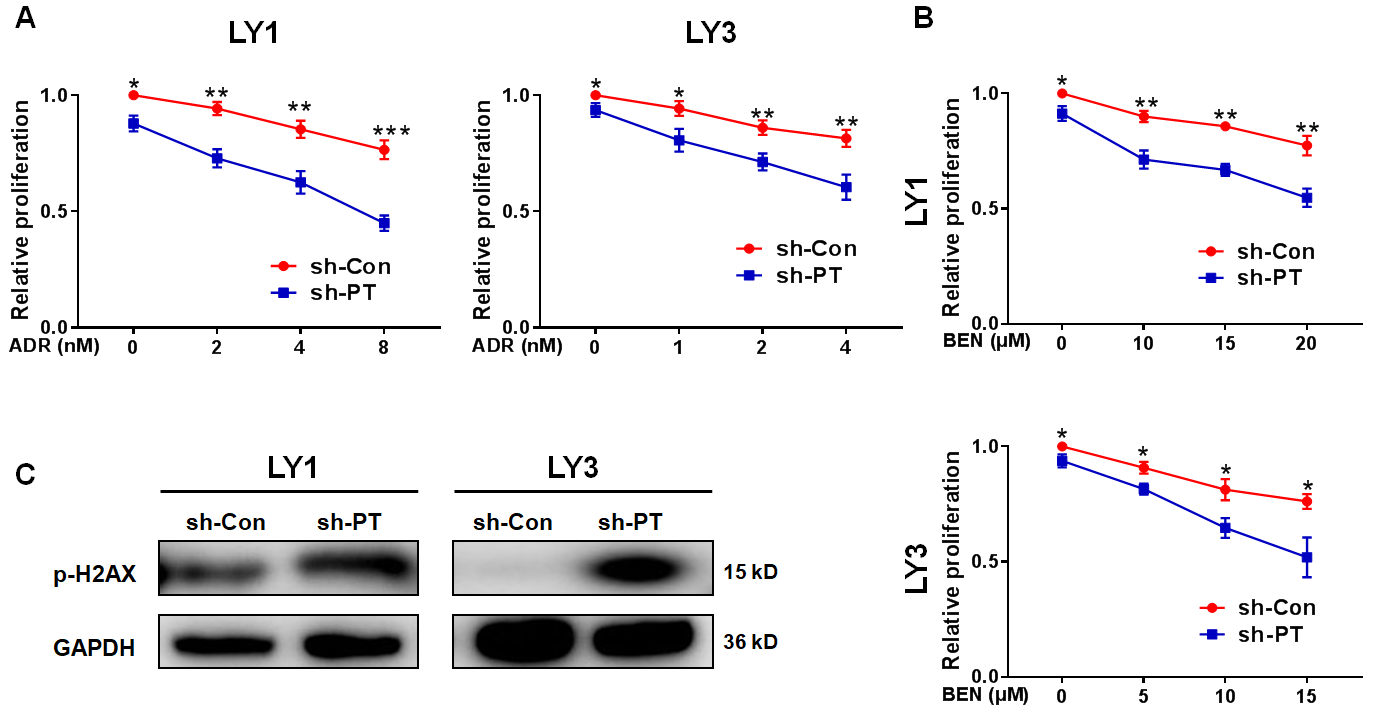


**Supplemental Figure 4. PTGDS knockdown enhanced chemo-sensitivity of DLBCL cells through promoting DNA damage.** A-B. PTGDS knockdown enhanced the cytotoxicity of ADR and BEN in LY1 and LY3 cells. C. Western blotting found that PTGDS knockdown increased the expression of p-H2AX. Data are shown as the mean ± SD. *p < 0.05;**p < 0.01


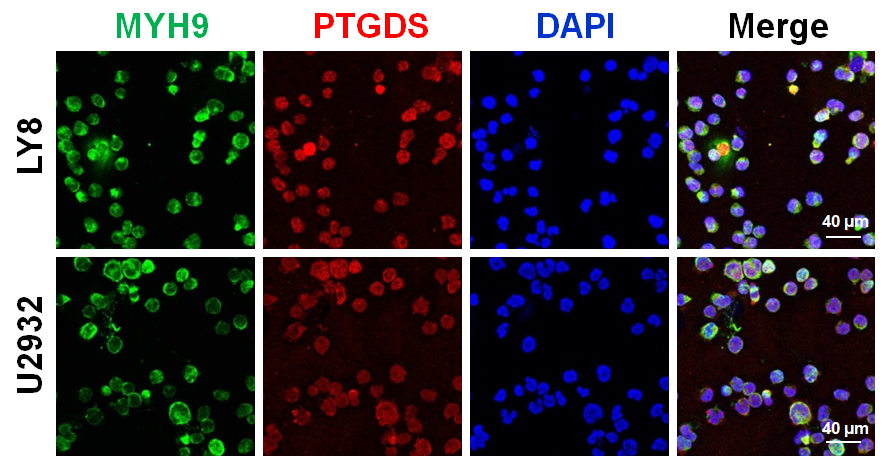


**Supplemental Figure 5. The colocalization of MYH9 and PTGDS protein in DLBCL**. Confocal immunofluorescent images indicated the colocalization of MYH9 and PTGDS protein in LY8 and U2932 cells. Bar = 40 μm.


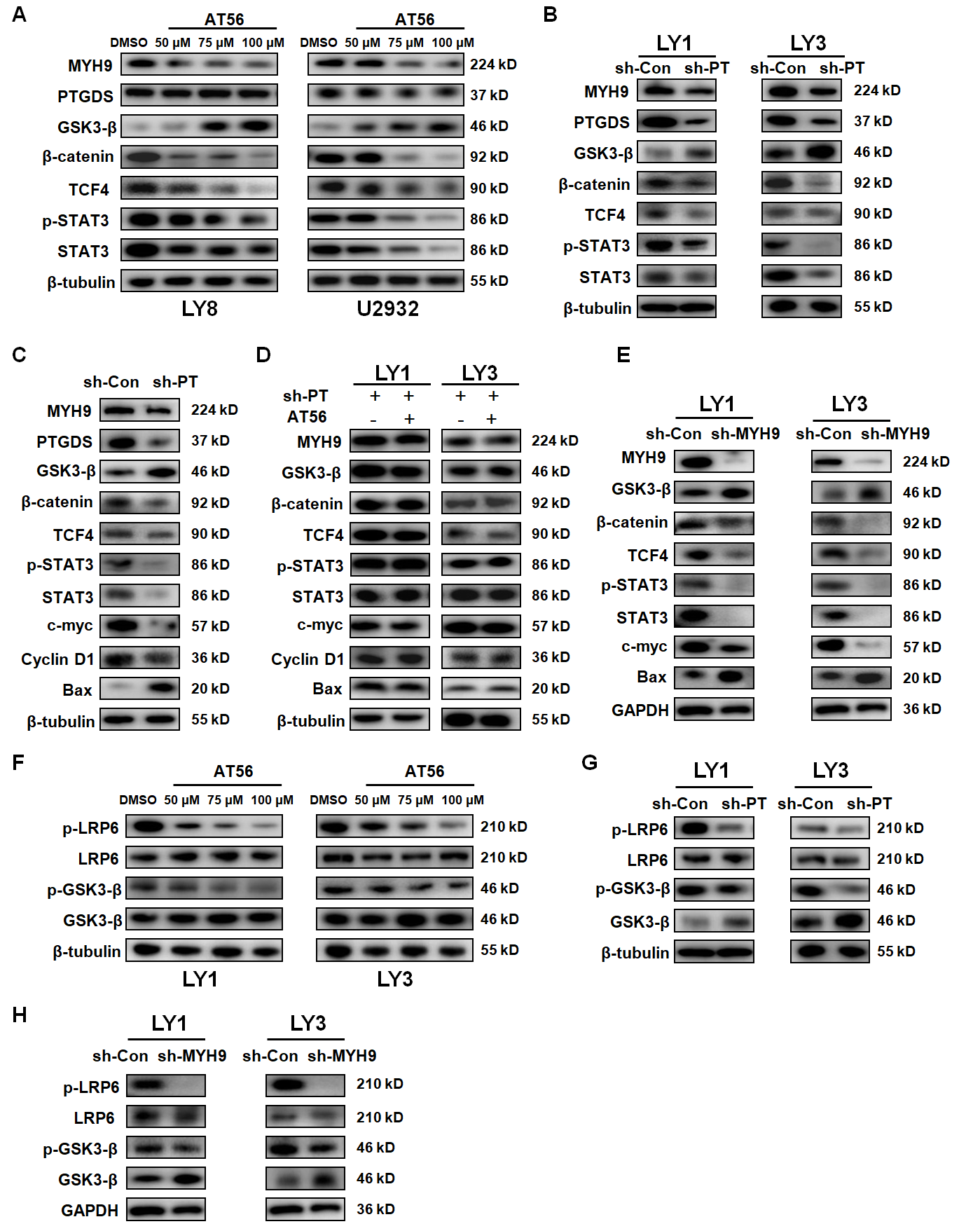


**Supplemental Figure 6. PTGDS inhibition and MYH9 knockdown** **suppressed the activation of Wnt-β-catenin-STAT3 signaling.** A. AT56 inhibited the expression of MYH9 and the activation of Wnt-β-catenin-STAT3 signaling in LY8 and U2932 cells. B-C. PTGDS knockdown inhibited the expression of MYH9 and the activation of Wnt-β-catenin-STAT3 signaling *in vitro* and *in vivo*. D. Western blotting analysis of MYH9 expression and activation of Wnt-β-catenin-STAT3 signaling after AT56 treatment in sh-PTGDS cells. E. MYH9 knockdown inhibited the activation of Wnt-β-catenin-STAT3 signaling in LY1 and LY3 cells. F-H. PTGDS inhibition and MYH9 knockdown reduced the phosphorylation of LRP6 and GSK3-β in DLBCL cells.

**
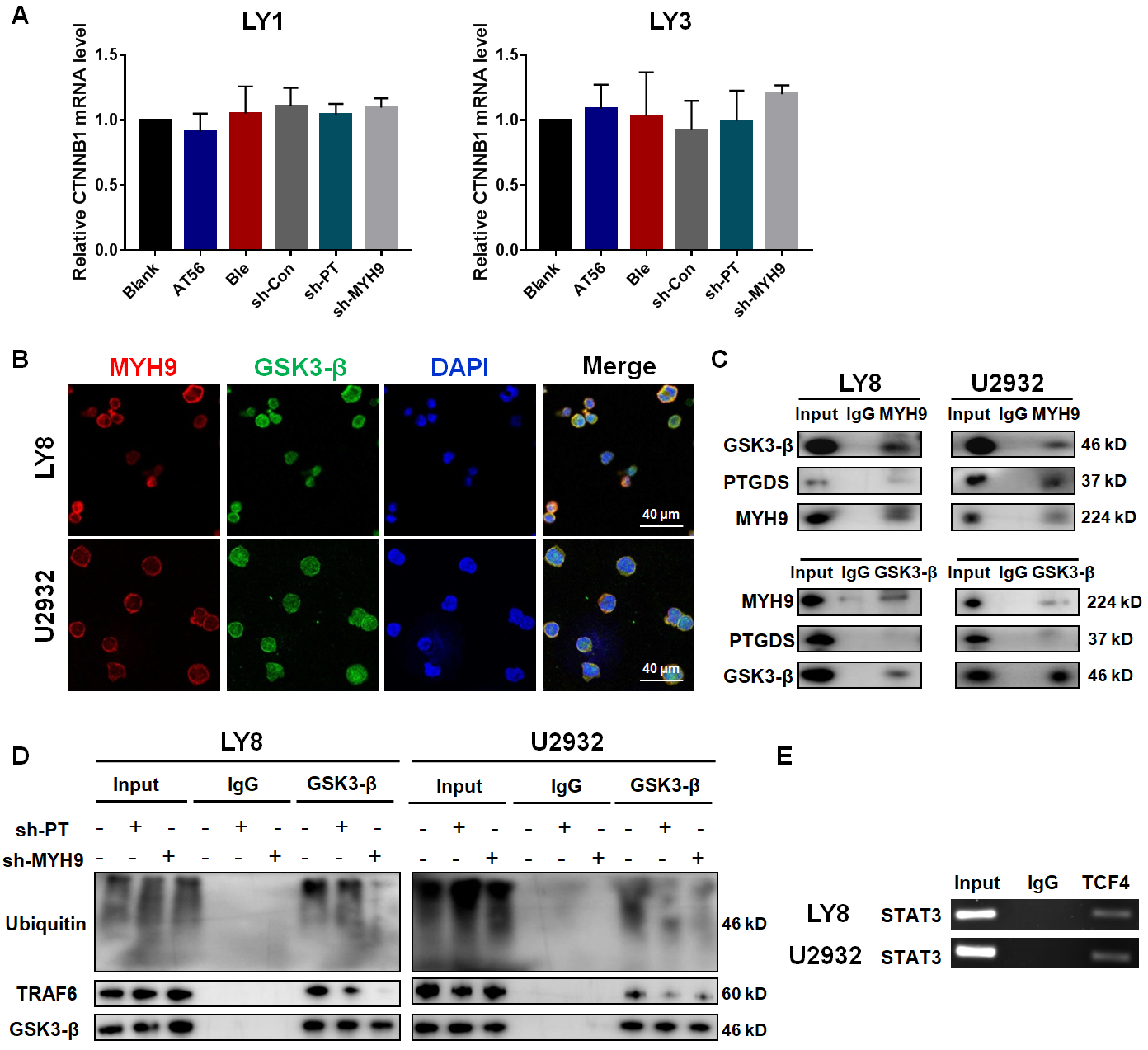
**

**Supplemental Figure 7. PTGDS and MYH9 regulated** Wnt-β-catenin-STAT3 **signaling through influencing the ubiquitination of GSK3-β in DLBCL.** A. The role of PTGDS and MYH9 inhibition on the expression of β-catenin mRNA in LY1 and LY3 cells. B. Confocal immunofluorescent images indicated the colocalization of MYH9 and GSK3-β protein in LY8 and U2932 cells. Bar = 40 μm. C. CoIP assay showed the bindings between MYH9 protein and GSK3-β protein in LY8 and U2932 cells. D. PTGDS and MYH9 inhibition decreased the ubiquitination of GSK3-β in LY8 and U2932 cells. E. ChIP assay found the binding between TCF4 and the promoter of STAT3 in LY8 and U2932 cells.

**
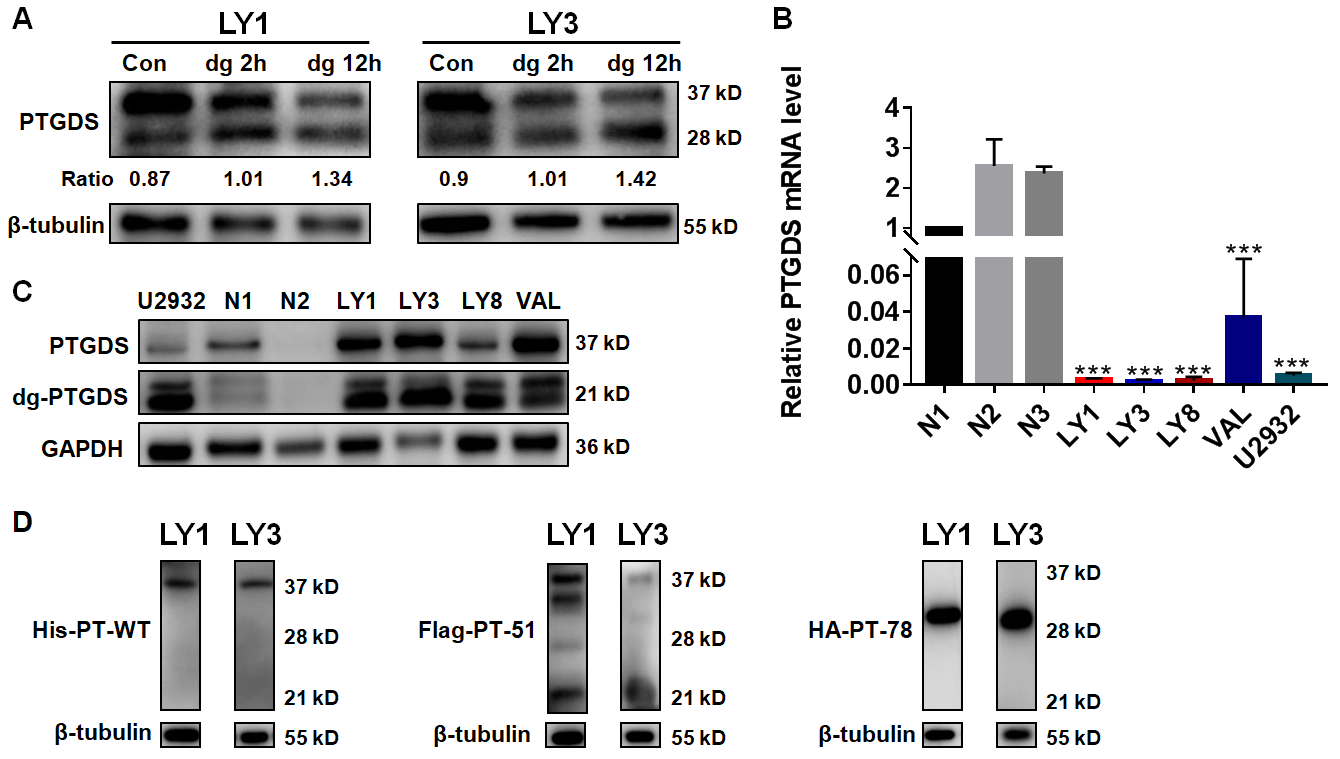
**

**Supplemental Figure 8.** **PTGDS protein in DLBCL cells was with low degree glycosylation.** A. The treatment of PNGase F (2h and 12h) increased the ratio of low molecular weight PTGDS protein. B. RT-PCR assays showed the low expression of PTGDS mRNA in DLBCL cells in comparison with normal B cells. C. Western blotting showed the level of PTGDS glycosylation was lower in DLBCL cells than that in normal B cells. D. Western blotting showed the decreased molecule weight of PTGDS protein after glycosylation site mutation (His-PTGDS-WT, Flag-PTGDS-△51, HA-PTGDS-△78).
