# Supplementary material for: Glycoprotein PTGDS promotes tumorigenesis of diffuse large B-cell lymphoma by MYH9-mediated regulation of Wnt–β-catenin–STAT3 signaling
Source: Cell Death Differ. 2021 Nov 6;29(3):642–56. doi: 10.1038/s41418-021-00880-2 (PMC8901925; doi:10.1038/s41418-021-00880-2)
Supplement: Supplementary file 3 — Supplemental Table 2 [file 41418_2021_880_MOESM3_ESM.docx]

**Supplemental Table 2.** Clinical characteristics based on PTGDS expression in DLBCL patients

| Characteristics | No. of  patients | Negative PTGDS  n (%) | Positive PTGDS  n (%) | P value |
| --- | --- | --- | --- | --- |
| **Age(years)** |  |  |  |  |
| <60 | 61 | 33（28%） | 28（24%） | 0.224 |
| ≥60 | 56 | 24（21%） | 32（27%） |  |
| **Gender** |  |  |  |  |
| Male | 56 | 27（22%） | 29（24%） | 0.845 |
| Female | 64 | 32（27%） | 32（27%） |  |
| **Ann Arbor Stage** |  |  |  |  |
| Ⅰ/Ⅱ | 43 | 18（17%） | 25（24%） | 0.287 |
| Ⅲ/Ⅳ | 63 | 33（31%） | 30（28%） |  |
| **IPI score > 3** |  |  |  |  |
| No | 61 | 31（30%） | 30（30%） | 0.286 |
| Yes | 40 | 16（16%） | 24（24%） |  |
| **Subtype** |  |  |  |  |
| GCB | 42 | 14（12%） | 28（23%） | **0.011** |
| Non-GCB | 78 | 45（38%） | 33（27%） |  |
| **Elevated** β**2-MG** |  |  |  |  |
| Yes | 19 | 7（7%） | 12（12%） | 0.323 |
| No | 83 | 41（40%） | 42（41%） |  |
| **Elevated** **Sialic Acid** |  |  |  |  |
| Yes | 21 | 14（14%） | 7（7%） | **0.048** |
| No | 80 | 34（34%） | 46（45%） |  |
| **Double Expression** |  |  |  |  |
| Yes | 23 | 10（35%） | 13（45%） | 0.228 |
| No | 6 | 1（3%） | 5（17%） |  |
| **Therapeutic Efficacy** |  |  |  |  |
| CR+PR | 32 | 19（31%） | 13（21%） | **0.013** |
| SD+PD | 29 | 8（13%） | 21（35%） |  |

Abbreviations: IPI, international prognostic index; MG, microglobulin; CR, complete remission; PR, partial remission; SD, stable disease; PD, progressive disease.
