# Supplementary material for: Glycoprotein PTGDS promotes tumorigenesis of diffuse large B-cell lymphoma by MYH9-mediated regulation of Wnt–β-catenin–STAT3 signaling
Source: Cell Death Differ. 2021 Nov 6;29(3):642–56. doi: 10.1038/s41418-021-00880-2 (PMC8901925; doi:10.1038/s41418-021-00880-2)
Supplement: Supplementary file 5 — Supplemental Table 4 [file 41418_2021_880_MOESM5_ESM.docx]

**Supplemental Table 4.** PTGDS associated proteins have been involved in the activation of Wnt pathway.

| ID | Gene | MW | Abundance | Unique Pep | UPC | PSM | Wnt pathway correlation |
| --- | --- | --- | --- | --- | --- | --- | --- |
| A0A024R1N1 | MYH9 | 226.4 | 464136004 | 48 | 27 | 55 | (1-3) |
| A0A087WUZ3 | SPTBN1 | 274.7 | 13732664 | 6 | 3 | 6 | (4) |
| A0A0S2Z3G9 | ACTN4 | 104.8 | 12582116 | 6 | 9 | 6 | (5) |
| A0A0S2Z428 | KRT6A | 60 | 12128195 | 1 | 22 | 16 | (6, 7) |
| A0A2R8Y6G6 | ENO1 | 47.3 | 7123287 | 3 | 8 | 3 | (8) |
| P05109 | S100A8 | 10.8 | 5166474 | 1 | 12 | 1 | (9, 10) |
| P00338 | LDHA | 36.7 | 4833342 | 2 | 12 | 3 | (11) |
| P21333 | FLNA | 280.6 | 1634269 | 2 | 1 | 2 | (12-14) |
| A0A5F9ZHM4 | LDHB | 37.4 | 1608018 | 2 | 12 | 3 | (15) |
| A0A161I202 | LTF | 78.3 | 1266684 | 1 | 1 | 1 | (16-21) |
| Q6UWP8 | SBSN | 60.5 | 1198553 | 3 | 18 | 4 | (22) |

MW: molecular weight; UPC: Unique Peptides coverage; PSM: peptide spectrum matched to the protein.

**Reference**

1. Liu L, Ning Y, Yi J, Yuan J, Fang W, Lin Z, et al. miR-6089/MYH9/beta-catenin/c-Jun negative feedback loop inhibits ovarian cancer carcinogenesis and progression. Biomed Pharmacother. 2020;125:109865.

2. Li YQ, Chen Y, Xu YF, He QM, Yang XJ, Li YQ, et al. FNDC3B 3'-UTR shortening escapes from microRNA-mediated gene repression and promotes nasopharyngeal carcinoma progression. Cancer Sci. 2020;111(6):1991-2003.

3. Ye G, Yang Q, Lei X, Zhu X, Li F, He J, et al. Nuclear MYH9-induced CTNNB1 transcription, targeted by staurosporin, promotes gastric cancer cell anoikis resistance and metastasis. Theranostics. 2020;10(17):7545-60.

4. Zhi X, Lin L, Yang S, Bhuvaneshwar K, Wang H, Gusev Y, et al. betaII-Spectrin (SPTBN1) suppresses progression of hepatocellular carcinoma and Wnt signaling by regulation of Wnt inhibitor kallistatin. Hepatology. 2015;61(2):598-612.

5. Wang Q, Qin Q, Song R, Zhao C, Liu H, Yang Y, et al. NHERF1 inhibits beta-catenin-mediated proliferation of cervical cancer cells through suppression of alpha-actinin-4 expression. Cell Death Dis. 2018;9(6):668.

6. Chen C, Shan H. Keratin 6A gene silencing suppresses cell invasion and metastasis of nasopharyngeal carcinoma via the betacatenin cascade. Mol Med Rep. 2019;19(5):3477-84.

7. Cui CY, Klar J, Georgii-Heming P, Frojmark AS, Baig SM, Schlessinger D, et al. Frizzled6 deficiency disrupts the differentiation process of nail development. The Journal of investigative dermatology. 2013;133(8):1990-7.

8. Mutze K, Vierkotten S, Milosevic J, Eickelberg O, Konigshoff M. Enolase 1 (ENO1) and protein disulfide-isomerase associated 3 (PDIA3) regulate Wnt/beta-catenin-driven trans-differentiation of murine alveolar epithelial cells. Dis Model Mech. 2015;8(8):877-90.

9. Duan L, Wu R, Ye L, Wang H, Yang X, Zhang Y, et al. S100A8 and S100A9 are associated with colorectal carcinoma progression and contribute to colorectal carcinoma cell survival and migration via Wnt/beta-catenin pathway. PLoS One. 2013;8(4):e62092.

10. van den Bosch MH, Blom AB, Schelbergen RF, Vogl T, Roth JP, Sloetjes AW, et al. Induction of Canonical Wnt Signaling by the Alarmins S100A8/A9 in Murine Knee Joints: Implications for Osteoarthritis. Arthritis Rheumatol. 2016;68(1):152-63.

11. Liu Y, Guo JZ, Liu Y, Wang K, Ding W, Wang H, et al. Nuclear lactate dehydrogenase A senses ROS to produce alpha-hydroxybutyrate for HPV-induced cervical tumor growth. Nat Commun. 2018;9(1):4429.

12. Lian G, Dettenhofer M, Lu J, Downing M, Chenn A, Wong T, et al. Filamin A- and formin 2-dependent endocytosis regulates proliferation via the canonical Wnt pathway. Development. 2016;143(23):4509-20.

13. Lian G, Chenn A, Ekuta V, Kanaujia S, Sheen V. Formin 2 Regulates Lysosomal Degradation of Wnt-Associated beta-Catenin in Neural Progenitors. Cereb Cortex. 2019;29(5):1938-52.

14. Adams M, Simms RJ, Abdelhamed Z, Dawe HR, Szymanska K, Logan CV, et al. A meckelin-filamin A interaction mediates ciliogenesis. Hum Mol Genet. 2012;21(6):1272-86.

15. Mazzio E, Badisa R, Mack N, Cassim S, Zdralevic M, Pouyssegur J, et al. Whole-transcriptome Analysis of Fully Viable Energy Efficient Glycolytic-null Cancer Cells Established by Double Genetic Knockout of Lactate Dehydrogenase A/B or Glucose-6-Phosphate Isomerase. Cancer Genomics Proteomics. 2020;17(5):469-97.

16. Huang HC, Lin H, Huang MC. Lactoferrin promotes hair growth in mice and increases dermal papilla cell proliferation through Erk/Akt and Wnt signaling pathways. Arch Dermatol Res. 2019;311(5):411-20.

17. Liu J, Li B, Lee C, Zhu H, Zheng S, Pierro A. Protective effects of lactoferrin on injured intestinal epithelial cells. J Pediatr Surg. 2019;54(12):2509-13.

18. Liu J, Zhu H, Li B, Robinson SC, Lee C, O'Connell JS, et al. Lactoferrin Reduces Necrotizing Enterocolitis Severity by Upregulating Intestinal Epithelial Proliferation. Eur J Pediatr Surg. 2020;30(1):90-5.

19. Amini AA, Nair LS. Recombinant human lactoferrin as a biomaterial for bone tissue engineering: mechanism of antiapoptotic and osteogenic activity. Adv Healthc Mater. 2014;3(6):897-905.

20. Reznikov EA, Comstock SS, Yi C, Contractor N, Donovan SM. Dietary bovine lactoferrin increases intestinal cell proliferation in neonatal piglets. J Nutr. 2014;144(9):1401-8.

21. Jiang R, Lonnerdal B. Transcriptomic profiling of intestinal epithelial cells in response to human, bovine and commercial bovine lactoferrins. Biometals. 2014;27(5):831-41.

22. Zhu J, Wu G, Li Q, Gong H, Song J, Cao L, et al. Overexpression of Suprabasin is Associated with Proliferation and Tumorigenicity of Esophageal Squamous Cell Carcinoma. Sci Rep. 2016;6:21549.
